# A Survey of Robotic Systems for Nursing Care

**DOI:** 10.3389/frobt.2022.832248

**Published:** 2022-04-07

**Authors:** Celia Nieto Agraz, Max Pfingsthorn, Pascal Gliesche, Marco Eichelberg, Andreas Hein

**Affiliations:** ^1^ R&D Department Production, OFFIS-Institute for Information Technology, Oldenburg, Germany; ^2^ Assistance Systems and Medical Device Technology, Department of Health Services Research, Carl von Ossietzky University Oldenburg, Oldenburg, Germany

**Keywords:** care, nursing, robot, human–robot interaction, automation, classification, survey

## Abstract

An increase of the aging population with a decrease in the available nursing staff has been seen in recent years. These two factors combined present a challenging problem for the future and has since become a political issue in many countries. Technological advances in robotics have made its use possible in new application fields like care and thus it appears to be a viable technological avenue to address the projected nursing labor shortage. The introduction of robots in nursing care creates an active triangular collaboration between the patient, nurse, and robot, which makes this area significantly different from traditional human–robot interaction (HRI) settings. In this review, we identify 133 robotic systems addressing nursing. We classify them according to two schemes: 1) a technical classification extended to include both patient and nurse and 2) a novel data-derived hierarchical classification based on use cases. We then analyze their intersection and build a multidimensional view of the state of technology. With this analytical tool, we describe an observed skew of the distribution of systems and identify gaps for future research. We also describe a link between the novel hierarchical use case classification and the typical phases of nursing care from admission to recovery.

## 1 Introduction

Recent advances in the general field of robotics, such as compliant collaborative robot design (Wolf et al., 2016; Piazza et al., 2019), real-time 3D perception (Rusu, 2010; Correll et al., 2016), autonomy (Tanwani and Calinon, 2016; Pérez-D’Arpino and Shah, 2017), social navigation (Charalampous et al., 2017), task space, and force control (He et al., 2020), facilitate the novel application domains where robotic solutions were previously not possible (Siciliano and Khatib, 2016). This especially holds for the nursing care domain, where robots are not only operated by one person, but two people are involved (nurses and patients) and one person is often the object of manipulation (usually the patient). In addition, in some cases, the patient manipulates himself or herself with the help of a robotic system. Robotic nursing care is an emerging field, which enjoys global attention in various research projects and some commercial systems.

An increase in the aging population, combined with the shortage of nursing staff, makes the increasing need for care one of the main humanitarian challenges of the future (Rothgang et al., 2012). Innovative technologies seem like a promising avenue in addressing this problem, and using them in care has been promoted in the last years. Robots can be found as one of these technologies that addresses the challenges of the unique human–machine interaction required to overcome this shortage problem (Hülsken-Giesler, 2015).

Our analysis is guided by the care process and its specific requirements on robotic systems. Textbook definitions of care have changed considerably in recent decades. Virginia Henderson characterized nursing in 1969 as follows: “The unique function of the nurse is to assist the individual, sick or well, in the performance of those activities contributing to health or its recovery (or to a peaceful death) that he would perform unaided if he had the necessary strength, will, or knowledge. And to do this in such a way as to help him gain independence as rapidly as possible. This aspect of her work, this part of her function, she initiates and controls; of this she is master” (Roberts and Henderson, 1996).

More recent definitions place a stronger emphasis on the coordinating activities and the overall responsibility of care: “Nursing encompasses autonomous and collaborative care of individuals of all ages, families, groups, and communities, sick or well, and in all settings. Nursing includes the promotion of health, prevention of illness, and the care of ill, disabled, and dying people. Advocacy, promotion of a safe environment, research, participation in shaping health policy and in patient and health systems management, and education are also key nursing roles” (International Council of Nurses, 2002).

Both definitions emphasize the great importance of interpersonal interaction and communication, or the process of building and maintaining the relationship between a patient and nurse. A disturbance of the negotiation of nursing support, as well as of the intersubjective understanding between the nurse and the person to be cared for by means of aids, should be avoided in any case.

In Johansson-Pajala et al. (2020), we found a definition of care robots that matches our understanding, the authors say: “we refer to care robots as machines that operate partly or fully autonomously with the aim of supporting potential users, older adults and relatives, as well as professional caregivers, in providing physical, cognitive, or emotional support.”

Krick et al. (2019) showed that the use of robots in care can be acceptable. However, even though there is a desire to increase the use of these new technologies and the presence of robots, it is important to ensure that the human is still at the center of the collaboration. Human–robot interaction (HRI) is about people, and the use of robotics in care does not aim at replacing the nursing staff, but at supporting and helping them. In order to have a successful HRI, it is necessary to fulfill certain requirements. First, the interaction has to be physically close and safe, so it is necessary to consider the physical contact between the robot and the user when designing a solution, in order to mitigate possible injures. Secondly, there is a dependable physical interaction in a shared workspace, and for this reason, the human’s intention and preferences have to be taken into account, so there is an interaction behavior and a realization of human-friendly motions (Gliesche et al., 2020).

**FIGURE 1:**
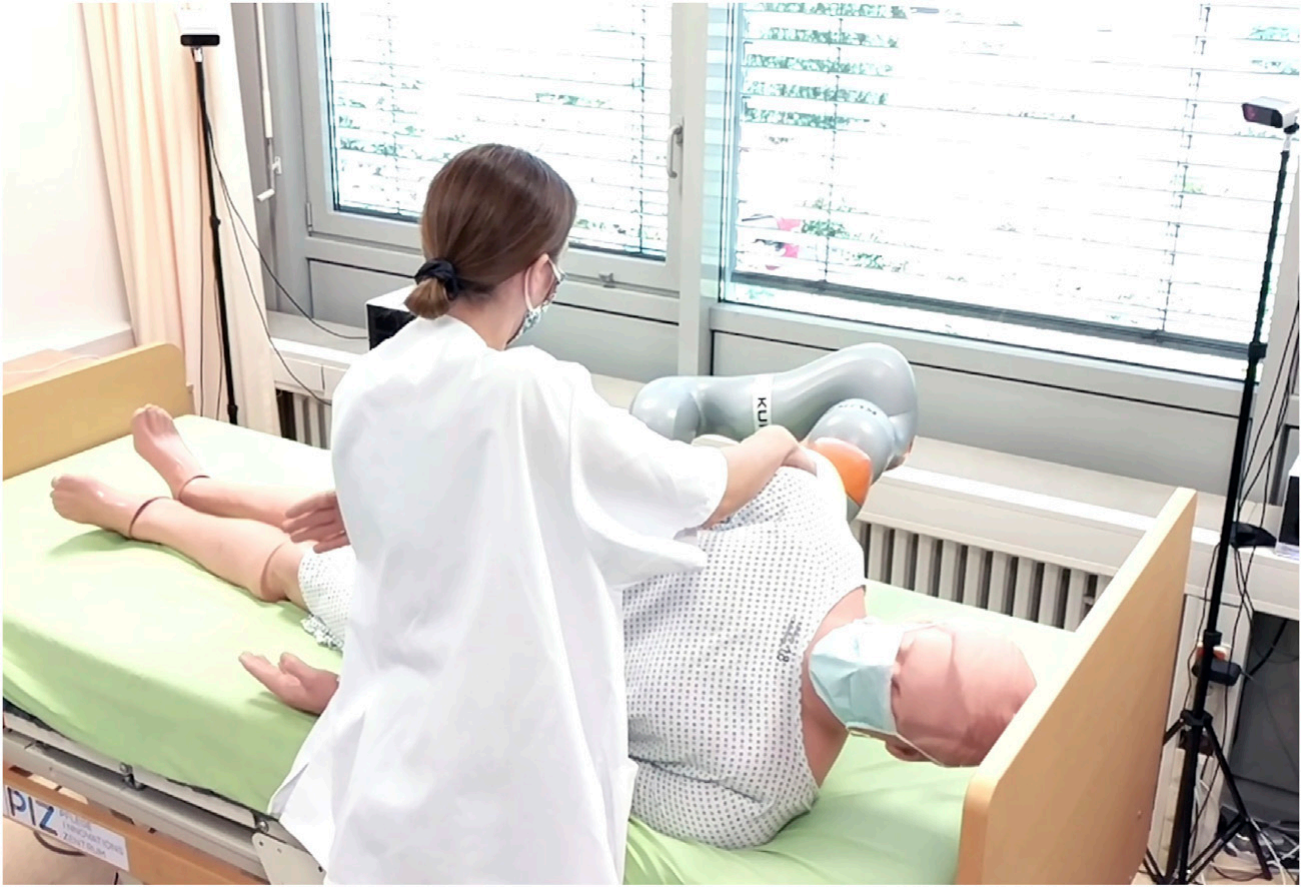
Scenario where the robot helps the nurse in the mobilization of the patient, easing some of the physical effort required for the action.

**FIGURE 2:**
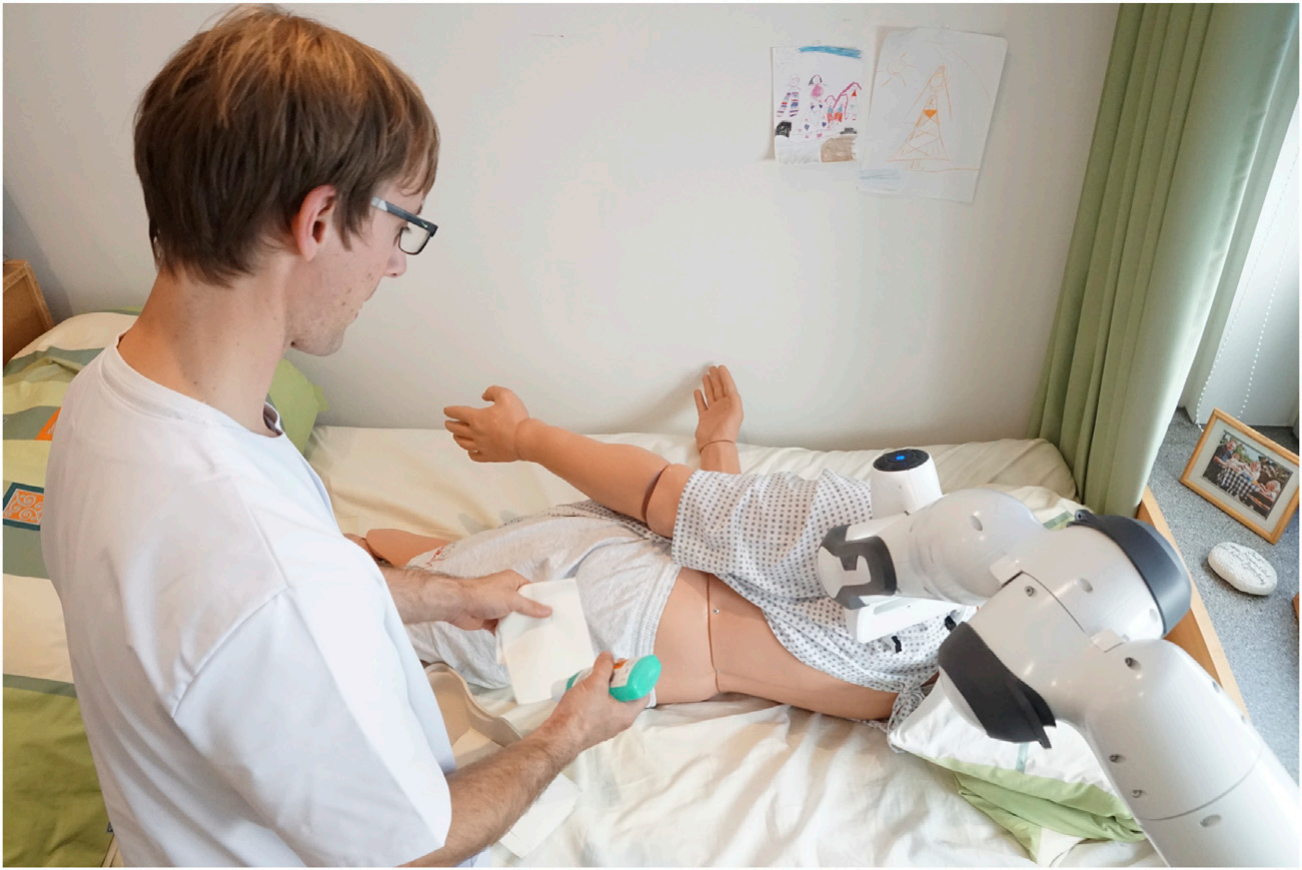
Scenario where the robot holds the patient in position, freeing both hands of the caregiver, so they can perform the care action (like cleaning the patient or healing a wound) faster and more agile.

**FIGURE 3:**
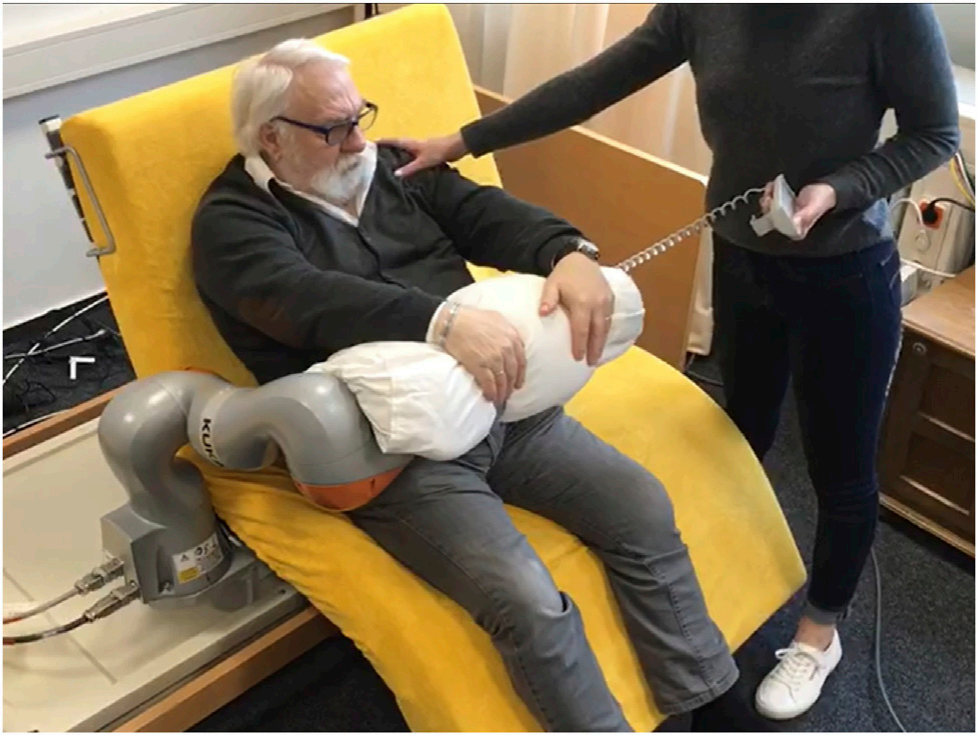
Scenario where the patient uses the robot as an assistance to get up without the help of the nurse.

**FIGURE 4:**
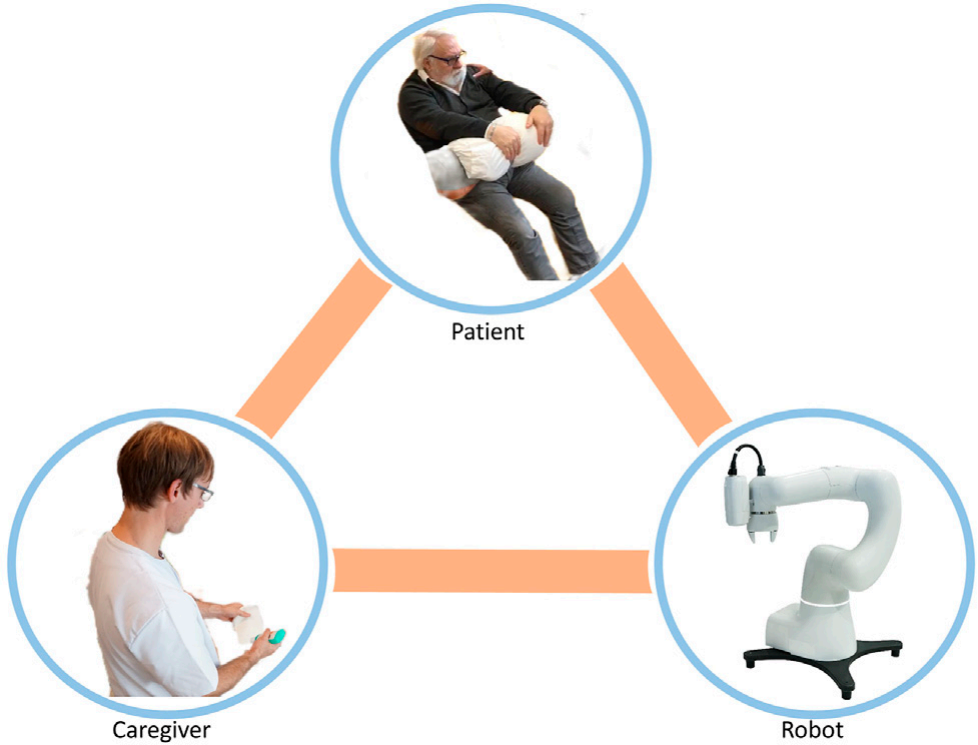
Schematic of the triangle relationship established among the patient, nurse, and robot.

In addition to these aspects of the use of robots in care, the following analysis is based on a triangular relationship between (professional or informal) caregivers, the person in need of care, and a supportive robotic system. Depending on the functional (sensory, cognitive, motor) limitations of the patient and the activities to be supported, the strength of the interaction between both the patient and their caregivers varies. Figures 1–3 show three different scenarios where this triangular relationship is presented, at the same time, Figure 4 schematizes it.

Kachouie et al. (2014) described how robots can benefit both the caregivers and patients. For the caregivers, the robots can help relieve them from tasks that are very time consuming and thus allow them to perform other tasks that are more useful and rewarding. For patients, the benefits are very extensive. Most of all, there is an increase of positive emotions, improving good feelings, general mood, and decreasing the stress and depression levels. They also promote engagement, increasing the commitment to physical activities and in also helping patients to externalize internal emotions. At the same time, there is an improvement in relationships, as they help with increasing social interactions and communication with other persons.

An important building block for this analysis is an overview of robotics projects in care, and a categorization of these according to a technical and a use case classification. Priority has been given to a broad coverage of projects worldwide. This provides an overview of the fields in care robotics where current investment in research and product development is concentrated and therefore could be considered as more relevant and to have bigger target groups. At the same time, this approach identifies fields that have not been addressed intensively yet and might provide potential for further study. A secondary aim of this review is also to provide these results in a way that can help caregivers and patients to identify which robotic technologies can be used for their specific use cases.

Due to the advances in the field of robotics and its possible use in nursing care, the aim of this review is to determine the directions in which the investigations in care robotics are going worldwide and to identify promising research and commercialization gaps. This article provides an overview of robots for care and projects that are prototyping them. It does not include projects that are developing or investigating applications of robots in care. After the identification of 133 projects worldwide, we performed an analysis of them, presenting the following contributions:

• A novel four-category classification, deduced from the consideration of the triangle robot, patient, and nurse in the technical classification from Haddadin and Croft (2016).
• An engineering-driven and actionable use case classification, defined by the authors, the first to our knowledge with this degree of specification in nursing care.
• A technical classification, directly obtained from Haddadin and Croft (2016).
• An analysis unifying the classification schemes above in a multidimensional view.
• A discussion of specific research and commercial opportunities resulting from the analysis.

## 2 Materials and Methods

We used the following resources and search terms to obtain a list of projects, as well as documentation that provides information about them.

### 2.1 Resources

We used three types of resources: online resources (databases), books, and conferences. The databases we used where the following: IEEExplore, Google Scholar, Springer Link, Robotics Today, Scopus, JSTOR, Science Direct, and CORDIS EU. Also, we used two books (Bendel, 2018) and (Hülsken-Giesler and Remmers, 2020), which contain previous analyses of robotics in care and define a list of robots in this field. Lastly, we used the database of the Federal Ministry of Education and Research (BMBF) to search for German projects.

### 2.2 Search Terms

**FIGURE 5:**
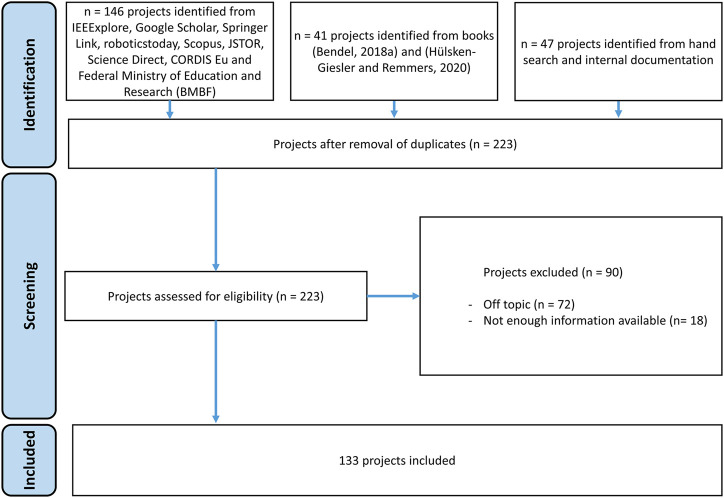
Search results and projects selection process.

We defined the following search string in several sessions. Due to the lack of comparable surveys, except the one from Krick et al. (2019), this inductive procedure had to be chosen. These terms allow for a broader scope, that only using those exclusively centered in care, as we wanted to make sure that we were able to include as much care-related projects as possible. As shown in Figure 5, we found a higher amount of projects, but we removed those that were out of our scope.

### 2.3 Search Strategy

(care OR caring OR care giving) AND (nursing OR nursing care) AND (elder care OR elderly care OR geriatric nursing OR geriatric care OR acute care OR older adult) AND (hospital OR inpatient care) AND (retirement home OR nursing home OR care home OR foster home) AND (long-term care OR short-term care OR home health care OR home nursing care OR home care OR home care nursing services) AND (formal caregiver OR professional care OR caregiver OR nurse) AND (professional caregiver OR geriatric nurse OR informal caregiver) AND (formal care OR family care OR informal care) AND (care recipient OR patient OR resident) AND (implementation OR application OR use OR case use OR usage OR utilization) AND (robot OR robotics OR robot system OR robotic system OR robot technology OR humanoid OR human robot OR humanoid robot OR human robotic) AND (human machine interaction) AND (service robot OR service robotic OR carebot OR carerobot OR) AND (autonom* robot*) AND (robot companions OR socio-assistive systems OR socio-assistive robots OR assistive robots) AND (geratronics OR gerontechnology) AND (emotional robot*).

### 2.4 Inclusion/Exclusion Criteria

In order to select the research articles, there was no limitation to the publication date, but because the search was done in May and June of 2020, we only included projects up to this date. We executed the search by means of the keywords above, but we selected documents according to the title, abstract, and full context, as long as these were relevant in the subject areas: care, robotics, and application. The type of article was not an exclusion criterion. We accepted professional magazine articles, essays, congress tapes, and reference books, as well as qualitative, quantitative, and mixed methods research work and reviews of all kinds, as long as there was access to the full text. We only considered documents written in English and German due to the available language skills in the research team. This article provides an overview of robots for care and projects that are prototyping them. It does not include projects that are developing or investigating applications of robots in care. Due to the fact that the search was not done by means of articles, but of products and projects, the articles found had to be read in order to select the ones those actually provide information about the robot system. Figure 5 shows the PRISMA flow diagram for the selection of the final projects included in this review.

## 3 Results

We identified a total of 133 relevant projects worldwide, including research and commercial products. We extracted 25 of these projects from internal documentation: 10 of them are the robotic projects that we supervise inside the BeBeRobot project (German Federal Ministry of Education and Research, 2022) (project within which this article is developed), and we got 15 from an internal project’s document. In both cases we only got the names of the projects from these lists and, we searched later for the documentation and information attached to them and shown in this article. As we explained, we focused our search on projects and products and not on systematic literature search, i.e., once we identified a project or product, we searched for further information that allowed us to classify and analyze them. Each project is at least published and described on a website, while for most of them, there are also popular and scientific articles that provide information about the project. The number of articles per project varies, but we selected a total of 161 articles. Supplementary Table S1 presents all the projects, along with the associated country and the specific references.

**FIGURE 6:**
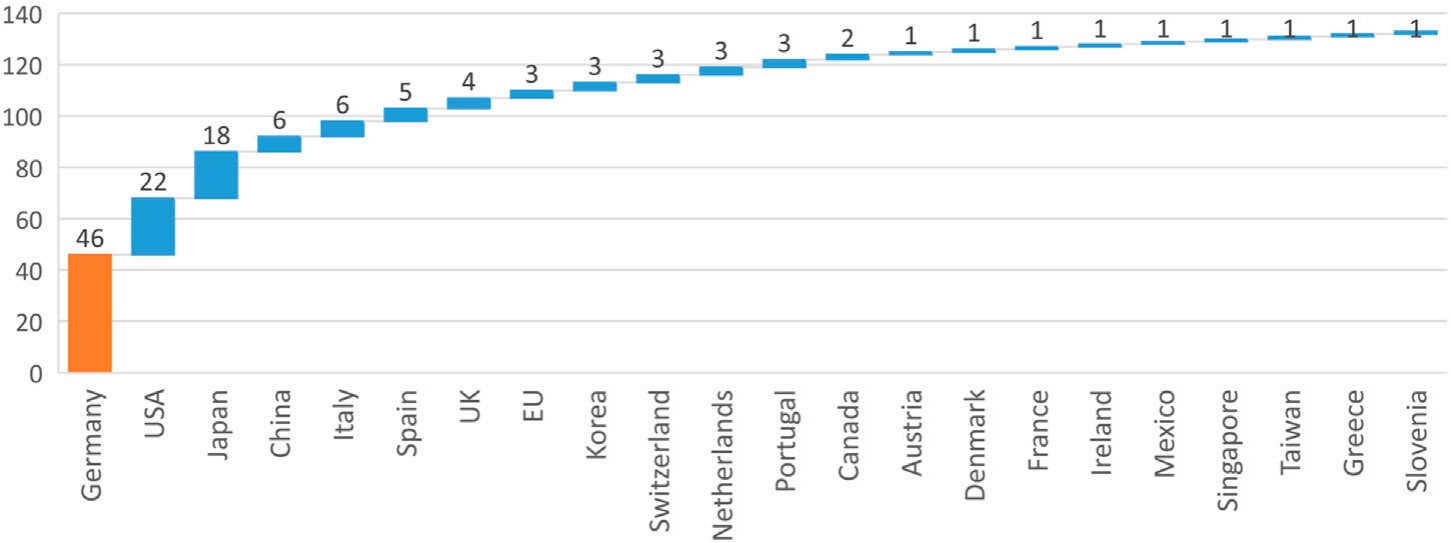
Number of projects found per country.

Figure 6 shows the number of projects found for each country. There is certainly a bias in the distribution of projects over countries because we could include German sources (in addition to English), which increases the number of German projects found, but we were unable to use similar sources in other languages.

The number of projects found in each country matched well with the distribution of articles across the countries included by Krick et al. (2019) in their scoping review on robotics in care. Only the number of projects found in Germany is significantly higher, since there were better search options (including the project database of the German Federal Ministry of Education and Research).

Among the international projects, it is possible to see that the two countries with a higher number of projects are the United Kingdom and Japan, both of which are large economic powers with significant capacity and need to invest in research and development of such new technologies and remain the largest markets for robotics after China (Bieller, 2019).

### 3.1 Technical Classification

**FIGURE 7:**
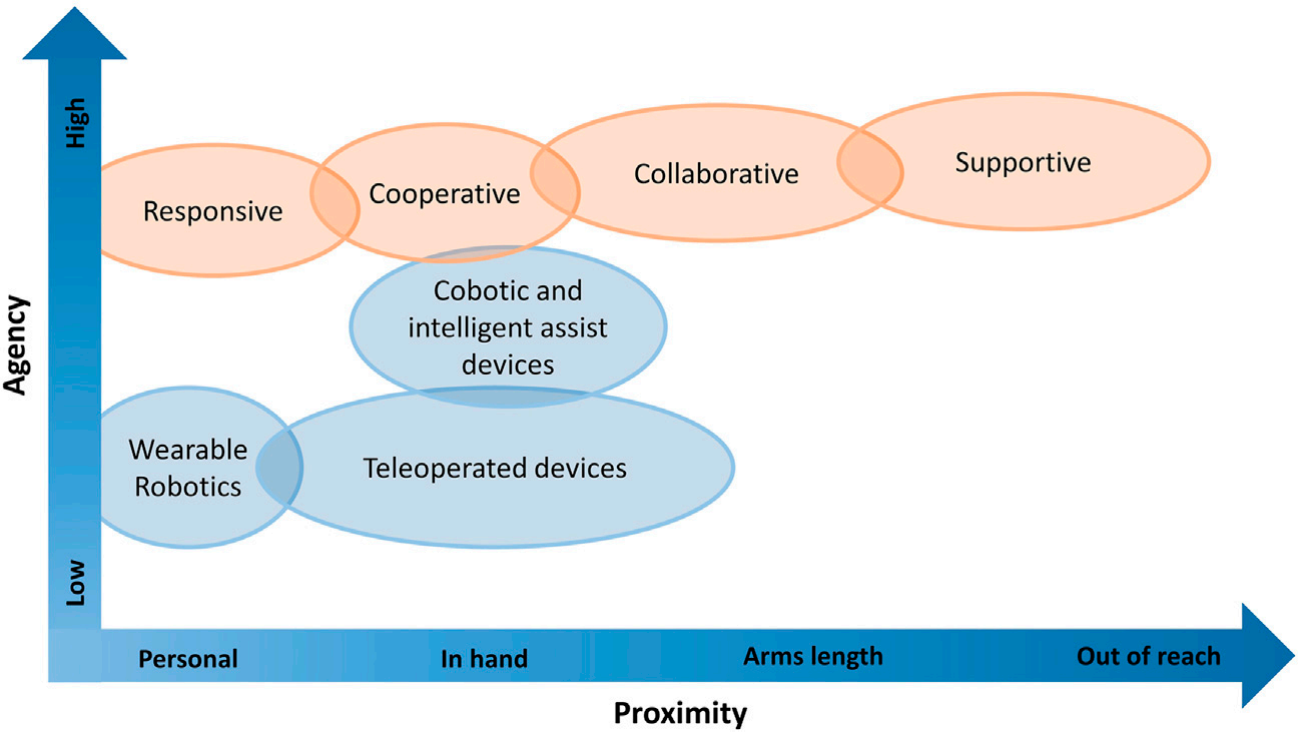
Classification scheme for HRI, by proximity of interaction and autonomy of the robot. Adapted from Haddadin and Croft (2016).

The first objective of this review is to identify a technical classification that allows a structured view of what kind of robotic technologies are available on the market or in research. A first scheme is extended from Haddadin and Croft (2016) (Figure 7) applied to the identified projects. Both parameters defined by them, proximity and autonomy, were assigned and used for classification. For proximity, we considered how close the interaction with the patient or caregiver is as two different dimensions. For autonomy, we considered to which degree the robot could act independently, or conversely, to which degree it was (remote) controlled. Low autonomy means that the robot needs to be controlled directly or receives significant input/supervision from the user. Medium denotes that at some point the robot requires some action from the user in order to proceed, or it requires that the user checks regularly if everything is working properly. High autonomy means that the robot does not have to be controlled by a user to operate at all.

Autonomy and decision-making are a very important aspect of assistive robots. According to ISO 8373 (ISO, 2021), a robot always has a certain degree of autonomy to perform its movements and tasks. However, the autonomy of some robots can go far beyond this. Kostavelis et al. (2017) showed a possible decision-making model in realistic situations, like those where service robots are designed to be used. Service robots can learn when to perform which task and when they need to take care of themselves (e.g., loading). This can lead to conflicts, especially in the medical and care sector. For example, unexpected situations can lead to a service robot actually having to charge its batteries at a time when it should be preparing critical medication. In this case, the robot must decide whether to perform the task that is important for the patient and then remain inactive or whether to charge first and deliver the medication to the patient too late, which may be time-critical, but still allow the robot to continue working. Alternatively, it would also be possible for the robot to outsource this decision to a human (nurse or patient). The German Ethics Council advocates “shared decision-making” here (Deutscher Ethikrat, 2020). In this way, the greatest good can be achieved for everyone involved. The adaptation of the service robots’ tasks and the degree of privacy can also be optimally adapted to the patient in this way.

In the following, the categories are described (see the Supplementary Material for details) by highlighting up to three examples of projects for each category. An exhaustive list of projects and their assigned categories are available in the Supplementary Material.

In the category **Supportive** (where robots assist in the performance of the task, providing tools or information), we find robots like TUG (Mutlu and Forlizzi, 2008; Niechwiadowicz and Khan, 2008; Zhang et al., 2008; AETHON, 2020), a robot to perform logistics activities in hospitals; BUDDY (Buddy Robotics, 2020), which has multiple functionalities at home (entertainment, monitoring old people, and reminding tasks and events); and AuRoRoll (German Federal Ministry of Education and Research, 2017; Wimmer et al., 2017) (when in automatic mode) a wheelchair capable of navigating autonomously. The parameters “Proximity” and “Autonomy” for these robots are identified as follows:

#### Proximity

• TUG: As it is a product designed to help nurses, patients have no contact with it, so we do not analyze the proximity aspect from their point of view. From the caregiver’s perspective, it is possible to define it as “out of reach”, even though there are moments when it comes closer (when delivering products), but most of the time, it moves autonomously, at a distance from the medical personnel.
• BUDDY: In this case, the situation is the other way around. Caregivers have barely any contact with the robot, at the most, only when checking on the elderly via the telepresence system, so we can define the proximity as “out of reach”. This is also the case from the user perspective, although in situations like playing it can also be considered “at arm length”, because of the closer interaction.
• AuRoRoll: In this case, the caregiver has also no role, but the patient can be considered in “personal” proximity, as they sit on the chair. Although the proximity does not correspond to the characteristic of this category, all the other characteristics of this project justify the classification.

#### Autonomy

In the three cases, the autonomy of the robots is “high”, as the three of them are able to drive and perform the different tasks autonomously and away from the user, once the action is required.

In the category **Cooperative** (where the human and robot share the control of the task in a continuous and cooperative way), example projects are: Care Robot Yurina (Robotics today, 2009; TechCrunch, 2010), which is designed to help in the lifting and transportation of patients or to be used as an electric wheelchair; ROBERT (Life Science robotics, 2020), a robot that takes over the repetitive task in rehabilitation situations; and Kaspar (Dautenhahn et al., 2009; Wainer et al., 2014a, 2014b; Huijnena et al., 2015; Wood et al., 2017; University of Hertfordshire, 2020) that works in therapy sessions with children with autism, with the objective to teach them different social skills.

#### Proximity

• Care Robot Yurina and ROBERT: from the patients' point of view, the proximity is “personal”, as they will have direct contact with the robot. From the caregiver’s point of view, it is “in hand” for Yurina, as they work together in the process of transferring the patient, and “in hand” or “arm length” for ROBERT, as they come into contact with the robot during the setting and the attachment process, as well as when indicating the exercise to perform.
• Kaspar: In this case, both the patient and caregiver are “in hand” or at “arm length” proximity: the patient (child) actively plays with the robot and the therapist supervises and leads the session.

#### Autonomy

In all three cases, the autonomy is high because the robots can perform the actions on their own. Nevertheless, it is a little bit lower than in the previous category, as they still need some control from the caregiver. In the case of the Care Robot Yurina, it gets input via voice or touchscreen for its control, for Robert, the nurse records the exercise and defines the number of repetitions and, when working with Kaspar, the therapist supervise the interaction between the robot and the user.

Projects such as Kinova Jaco (KINOVA, 2020) and Lio (Bendel, 2020; F&P Personal Robotics, 2020) can be classified in the **Collaborative** category. In this category, the human divides the task with the robot, and they perform the part that better suits them. The first example is a robotic arm that can be mounted on a wheelchair to assist people with limited or no upper limb mobility. Lio is a mobile personal robot with a multifunctional arm that can communicate with people and help them with their daily life activities, assisting healthcare professionals, or entertaining patients.

#### Proximity

• Kinova Jaco: the caregiver has no interaction with it, and they are released from tasks that they used to do, like picking up objects or feeding the patient. From the patient’s point of view, it can be considered as “in hand” or at “arm length” proximity, as it is mounted on the wheelchair, and the user can control it via a joystick or head control.
• Lio: in both cases of patient and nurse, the proximity can be defined as “in hand” or at “arm length”, as the robot interacts with them by giving objects or receiving requests, among others.

#### Autonomy

The autonomy of both robots is rather high, as both of them can perform the actions autonomously, once the user instructs them.

For the category **Responsive** (where interaction between this type of robots and the human (patient) is by means of touch), we found Paro (PARO Robots, 2014), a renowned example system in care that is designed to bring animal therapy to places where it is not possible to be administered with real animals. Huggable (Robotics today, 2006; Stiehl et al., 2006; Personal Robots Group, 2015; Jeong et al., 2018), which also aims at providing the benefits of animal therapy and helping the staff who work with the residents, and OurPuppet (Kuhlmann et al., 2018; Naroska et al., 2018; German Federal Ministry of Education and Research, 2019a; OurPuppet, 2019), which is a robot with no animal shape that tries to achieve similar benefits as the two previous examples. The main objective of the three robots is to reduce the stress and depression levels in users and to improve their emotional health and interaction capabilities with the other patients and the medical staff.

#### Proximity

• Paro and OurPuppet: in both cases, the caregiver has no role with the robot, but from the patient’s point of view, the proximity can be defined as “personal”, as the user holds the robot and very closely interacts with it.
• Huggable: in this case, the proximity with the patient is also considered “personal”, because of the very close interaction between the user and the robot. Furthermore, it can also be considered “out of reach” for the caregiver because they use the robot from a distance to communicate with the user and receive behavior data.

#### Autonomy

In the three cases, the autonomy of the robot can be considered as medium, as the robots perceive the user and their actions through different sensors and periodically learn how to interact with them, but still there is the need of a caregiver who supervises the interaction.

In the category **Wearable Robotics** (wearable devices, designed to be worn by humans), we have classified, for example, the exoskeleton Robot Suit Hal (Cyberdyne, 2020). This is a robot used for medical treatments of functional improvement of patients with cerebral, nervous, or muscle disorders, namely, spinal cord injury and cerebral embolism. ReWalk (Reddit Robotics, 2020) is a wearable exoskeleton with motors at the hip and knee joints, to enable people with spinal cord injury to stand up and walk. Lastly, we can also assign CareJack (German Federal Ministry of Education and Research, 2015a; Klinikum Stadt Soest, 2016; Kostelnik, 2016; Kuschan et al., 2016, 2017; Moritz and Hahn, 2016; Mularczyk, 2016; OTW, 2016; Wolschke et al., 2016) here, an exoskeleton, similar to a vest, which aims at supporting nurses in the mobilization of the patient.

#### Proximity

• Robot Suit Hal and Rewalk: from the patient’s point of view, it is “personal”, as it is attached to the patient’s body. From the caregiver’s point of view, it is at “arm length”, as they encounter the robot during the setting and the attachment process.
• CareJack: this case is the other way around, the patient acts more like an object in the interaction. But from the caregiver’s point of view, the proximity is classified as “personal” because it is attached to the body of the nurse.

#### Autonomy

Exoskeletons only assist the movement of their user, which means that the robot movement is based on an analysis of the biosignals that the body of the user emits. This means that the autonomy of the robots is low.

Projects like a Baxter-based dressing assistant (Gao et al., 2015, 2016; Zhang et al., 2017, 2019; Personal Robotics Lab, 2020), Pillo (Pillohealth, 2020), or AuRoRoll (in manual mode) (German Federal Ministry of Education and Research, 2017; Wimmer et al., 2017) can be classified in the category **Cobotic and intelligent auxiliary devices**. This kind of robots is designed to help the user perform daily life activities. Baxter is an assistive robot that, by means of a neural network, can assist disabled and elderly people in their daily dressing activities. Pillo, on the other hand, is a home assistance robot and an automated medication dispenser. Lastly, AuRoRoll (in manual mode) is a wheelchair that avoids obstacles and collisions while the user gives the directions.

#### Proximity

• Baxter-based dressing assistant: in the case of this robot, the nurse is out of the loop, so we do not analyze the proximity with them. From the patient’s point of view, the proximity can be classified as “in hand” or “arms length” because the robot has to be close enough to help them get dressed.
• Pillo: when considering the proximity with the patient, it can be defined as “in hand” because the user has to collect the medicines dispensed by it and read the notifications on its screen. As caregivers can remotely control the robot to receive notifications or manage some actions via the app, the proximity is considered “out of reach”.
• AuRoRoll: again in this case, the caregiver has no role in the interaction, and from the patient’s point of view, it can be considered as “personal” because the user sits on the chair and as “in hand” because the user uses the joystick to direct the chair.

#### Autonomy

In the case of Baxter, the autonomy is considered high, as it uses neural networks to learn how to optimally dress the person, which does not match the characteristic autonomy of this class, but for the rest of the characteristics, the authors believe that that this is the most representative class for this project. For the other two examples, it is considered that the autonomy is medium. In the case of Pillo, it has to be refilled and programmed to dispense the medicines at a personalized time, and it is also monitored by caregivers. Since AuRoRoll is considered in manual mode here, the user still provides all the directions and the robot only avoids collisions.

For the last class, **Teleoperated devices** (whose robots are remotely controlled), we identified TRINA (Li et al., 2017; Worcester Polytechnic Institute, 2022) and Ava (Robotics today, 2003; IEEE SPECTRUM, 2012). The first one is a tele-nursing robot designed to assist the nursing staff with communication and logistics tasks in quarantine areas, reducing the risk of exposure to infectious diseases. The second one is another telepresence robot that allows doctors to examine patients without being in the same room.

#### Proximity

• TRINA: with regard to the patient, the robot is at “arms length”, as it gives objects to them or takes their vital signs. Nevertheless, it could also be considered “out of reach”, if they are performing activities like taking away a dirty blanket or food tray. With regard to the caregiver, it is “out of reach”, as they control it over a distance to avoid entering the infected area.
• Ava: in this case, the patient is at “arms length”, as they have to be close enough for the examination. From the caregiver’s point of view, the robot is “out of reach”, for the doctor who remotely controls the robot.

#### Autonomy

**FIGURE 8:**
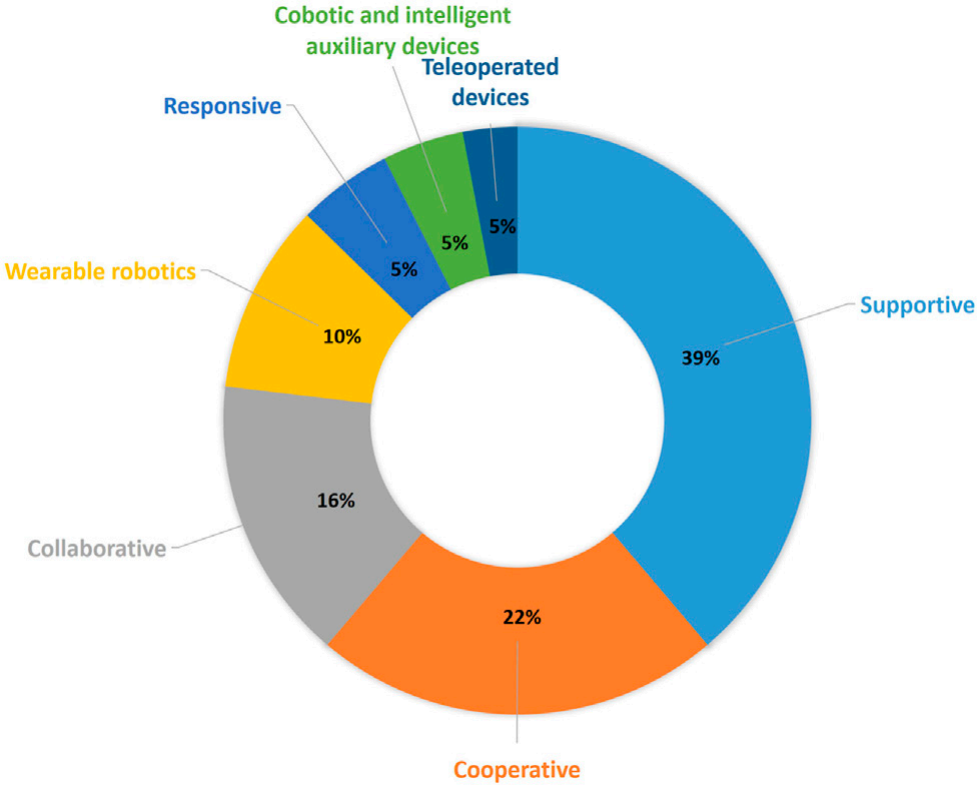
Percentage of project per each category of the technical classification.

The autonomy in these two examples is low because the care personnel controls the robot remotely. The distribution of the projects among the categories of the technical classification is shown in Figure 8. Interestingly, it can be seen that the three main categories for which the projects were found are *Supportive*, *Collaborative*, and *Cooperative*, covering almost 80% of the projects.

Because of the triangle presented in care (patient, nurse, and robot), we have to consider both the proximity of the robot to the patient and nurse, as we have shown in our analysis. Therefore, it is possible to modify the graph presented by Haddadin and Croft (2016) into a three-dimensional one. In this extended graph, we analyze for each degree of autonomy (low, medium, and high), the proximity of the robot with caregivers and patients.

**FIGURE 9:**
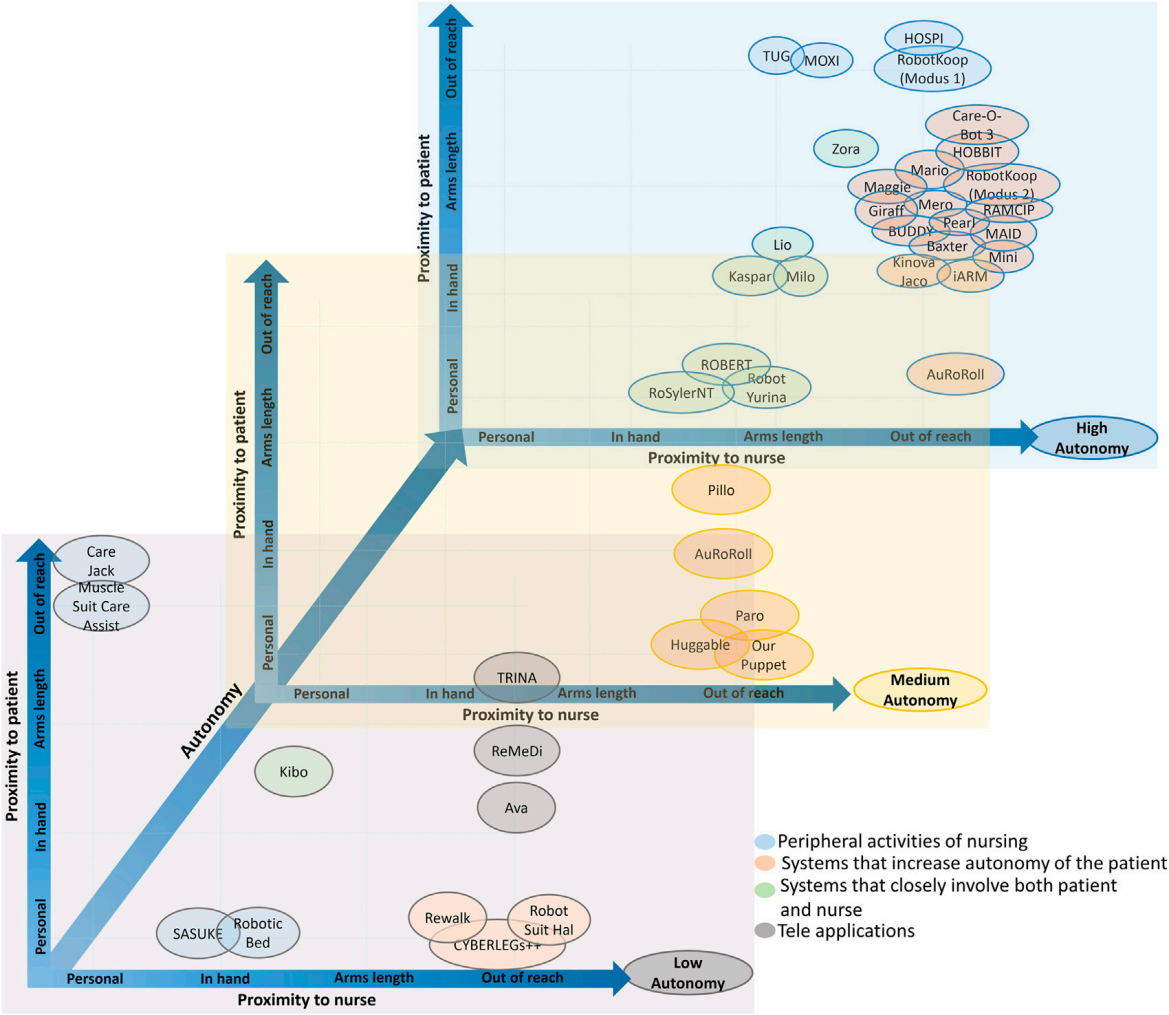
Distribution of example projects according to autonomy and proximity to patient and caregiver.

Figure 9 shows the distribution according to these three parameters (robot’s proximity to the nurse, robot’s proximity to the user, and autonomy of the robot) of the projects, which were given as examples and explained in the technical and use case classifications.

From Figure 9 we can deduce four categories:

• Peripheral activities of nursing: projects inside this category support the nurses away from the patient, thus allowing the nurse to focus on more important, patient-oriented tasks. They have little or no interaction with patients, and in cases where they do, the patient behaves more like an object receiving the action.
• Systems that increase autonomy of the patient: in this case, the caregivers are the ones with almost no involvement with the system, and the robot is designed to help and assist the patient.
• Systems that closely involve both patient and nurse: in this category, both the nurse and the patient interact directly and actively with the robot.

• Tele applications: Both the patient and the nurse are involved, but the caregiver is far away and they can remotely control the robot to interact with the patient over a distance.

### 3. 2 Use Case Classification

The second objective of this review is to identify the fields of application, or use cases, for which most robots are developed, which implies that these use cases are seen as the most desirable. At the same time, we wanted to identify the fields of application that are not covered well by existing robots or projects and may still offer room for future research and development. In order to do so, we define an engineering-driven and actionable use case classification. To create this classification, we use some of the classes (cleaning, toy robot, rehabilitation, logistics, diagnosis, companion robots, and personal aids and assisting devices) from Bulgheroni (2016), International Federation of Robotics (2016), Ben-Ari and Mondada (2018), and Krick et al. (2019) and complete this list with those classes that we considered missing and necessary after analyzing the projects. In addition, we map these classes to the four categories deduced from Figure 9. The final list of classes is explained in the Supplementary Material, and in the following paragraphs, we present two or three examples to illustrate them. The selected examples are those considered to best represent the category and, when possible, to those different to the ones presented in the technical classification, in order to show a higher variety.

#### 3.2.1 Peripheral activities of Nursing

• Logistics: robots that perform logistics chores. Good examples of this type of robots are HOSPI (Panasonic, 2015; Robotics today, 2015; Panasonic, 2019), MOXI (Dilgent Robotics, 2020), and Care-O-Bot 3 (Graf et al., 2009; Robotics today, 2010a; Fraunhofer, 2020). The first one is an autonomous vehicle that transports food, medicines, and medical supplies inside hospital environments. The second one is a robot that autonomously fetches and collects medical supplies for the nurses, saving their time. In addition, the latter transports and brings desired objects to the user at home.
• Transport of patients/transfer robot: they are used to lift and transport patients. Here, we can find projects like the Robotic Bed (Robotics today, 2010b; Panasonic, 2020), a robot providing the fusion of an electric care assistance bed with a wheeled chair; SASUKE (Muscle, 2020), which helps the nurse lift and transport patients; and the Muscle Suit Care Assist (Innophys, 2020), an exoskeleton that assists the movement and reduces strain when carrying other people. The objective of the above three is to prevent injuries caused by the physical effort required during the action of carrying.
• Cleaning: these robots are used to perform cleaning chores. One of the two examples found in this category is RobotKoop (German Federal Ministry of Education and Research, 2020a; Hochschule Ravensburg-weingarten, 2020; RobotKoop, 2020) that can be used in two scenarios: one at hospitals or public care areas with passers-by, where the robot can autonomously perform its task and communicate with people to coordinate its plan and support the cleaning at home. In this scenario, the robot can tidy up the apartment and rearrange known objects. In case of new elements, it will ask the user, learning from the input for future similar situations*.*

#### 3.2.2 Systems which Increase Autonomy of the Patient

• Companion robots: they are used to reduce feelings of isolation and loneliness in patients. Here, we can find project Mario (European Commission, 2019; Casey, 2019; eHealth Ireland, 2020), whose robot (Kompaii robot) allows people with dementia to access newspapers, provides reminders of upcoming events, allows users to listen to their favorite songs, helps them connect with their friends and families, and plays games for cognitive training. Maggie (C. I. U. M. RoboticsLab, 2014) talks to the user, reads magazines for them, or plays games to entertain and train cognitive functions. Finally, Pearl (Pollack et al., 2002) reminds the user to perform routine activities, such as eating, drinking, using the toilet, or taking medication, and it can as well guide the user through the environment.
• Personal aids and assisting devices: robots that provide help with daily life activities. In this group, very different kinds of robots can be found. There is, for example, iARM (Assistive innovations, 2020), an intelligent robot arm mounted on the user’s wheelchair that allows the user to grasp and manipulate objects from their surroundings. There is Pillo (Pillohealth, 2020), an automated medication dispenser that provides the right medication at the right time to the user and can be parameterized via an app by nurses and family. The last example is RAMCIP (Abdelnour et al., 2016; Peleka et al., 2018; European Commission, 2022), a service robot designed to assist people with Alzheimer with activities like cooking, eating, or medication ones. It can also communicate with the user and monitor them and their environment in order to act if necessary; lastly, the user can train their cognitive abilities by means of playing games with it.
• Mobility support: robots that help the patient to move. MAID (German Federal Ministry of Education and Research, 2015b; Guhl, 2015; Irgenfried and Schneider, 2016; Wagner, 2016) and CYBERLEGs++ (CYBERLEGs, 2020; Martini et al., 2020; European Commission, 2021) are examples of this kind of robot. They both help the user to stand up and walk. The former has different operating modes, from the robot being just a point of support for the user, to a mode where the robot helps the person to stand up, going through a mode where it can assist as a walking aid. CYBERLEGs++ on the other hand is a project, designed to test the viability of the powered robotic ortho-prosthesis in amputees. The prosthesis looks to improve and restore the mobility of amputees, in order to help them perform activities such as walking, climbing the stairs, or sitting up and down, and increasing their quality of life.
• Therapy support: these robots are used in therapy sessions. The most well-known example of this group is Paro (PARO Robots, 2014), a robot used in environments where animal therapy is not possible otherwise. As another example, we can find Milo (Hanson et al., 2009; Salvador et al., 2015; Hanson Robotics, 2020; Robtos.nu, 2020; Robtos4autism, 2020), a humanoid robot used in sessions with kids with autism, in order to teach them social skills such as tuning into emotions, expressing empathy, or acting more appropriately in social situations. Lastly, we can also find robots for physical or cognitive therapy, like Mini (Salichs et al., 2016), designed for old people suffering from Alzheimer’s disease, or other causes of cognitive impairment and, by means of games, it works with them and stimulates the different cognitive skills.
• Toy robots: they are used to distract and entertain the user. Two examples of this kind of robots are HOBBIT (Mayer and Panek, 2013; Vincze et al., 2014; Hobbit, 2020) and KIBO (Albo-Canals et al., 2018; Tufts University, 2018; González-González et al., 2019). The first one is a companion robot that has, among other abilities, the capacity of displaying multimedia content and games to entertain the user and work some cognitive skills. The second one is a robot designed to teach neurotypical children, as well as children with autism and Down syndrome to code (program) by means of games.

#### 3.2.3 Systems which Closely Involve both Patient and Nurse

• Rehabilitation: robots that help and assist in rehabilitation processes. In this group, we can find robots that are in direct contact with the patient, like Robot Suit Hal (Cyberdyne, 2020) and RoSylerNT (MEDICA Magazine, 2019; German Federal Ministry of Education and Research, 2020b; KIT, 2020), and those that are used to lead a rehabilitation session as an instructor, such as Zora (Vital et al., 2013, 2018; Pulido et al., 2017; Zorabots, 2020). Robot Suit Hal is an exoskeleton for patients with cerebral, nervous, and muscle disorders. The exoskeleton reads the bioelectric signals of the patient's body and by means of the control power unit assists movement. RoSylerNT is a learning rehabilitation system that is in direct contact with the patient and that actively applies forces and thus becomes an interactive training partner for the user. In both cases, the therapy adjusts the robot to the patient and is in charge of the cognitive part of the session. In the case of Zora, this robot can be used to lead the sessions along with the therapist, showing the user how the exercises have to be performed and correcting them when they do it wrong.
• Teaching robots: these robots aim at helping the user to develop a certain skill. It is common to find in this category of robots teaching social skills to children with autism, like the robot Kaspar (Dautenhahn et al., 2009; Wainer et al., 2014a, 2014b; Huijnena et al., 2015; Wood et al., 2017; University of Hertfordshire, 2020). This robot participates in the session with the therapist, helping the child explore basic emotions or enabling cognitive learning by playing games, designed to teach basic socially acceptable ways of interaction. Another example of this type is the robot Mero (Robotics Today, 2010c; Lee et al., 2010), a head-only robot, designed to teach English to children in Korea.

#### 3.2.4 Tele Applications

• Telepresence: these are remotely controlled robots that allow the user to be somewhere else. For example, the robot Kompaii in project Mario (European Commission, 2019; Casey, 2019; eHealth Ireland, 2020) is used to address the challenges of loneliness, isolation, and dementia in older persons, by means of games and video calls, allowing the user to be more in contact with their loved ones. Another example is Giraff (Coradeschi et al., 2013, 2014; Barsocchi et al., 2016), a robot that can be used to remotely control the health of the patient or to identify unusual situations at home, like falls. At the same time, it also offers the possibility of a virtual visit, which enables contact with relatives and caregivers.
• Diagnostic Systems/Telediagnosis: they allow the doctor to perform a diagnosis from a distance. Two examples of such robots are ReMeDi (Stollnberger et al., 2014; Arent et al., 2016; Center for Human-Computer Interaction, 2020) and Ava (Robotics today, 2003; IEEE SPECTRUM, 2012). They are both telepresence robots that allow the doctor to examine patients over long distances. The first one is specifically used for palpation and ultrasonographic examination, while the second one is used for examinations that are more general. In both cases, the doctor can teleconference and interact with the patient.

**FIGURE 10:**
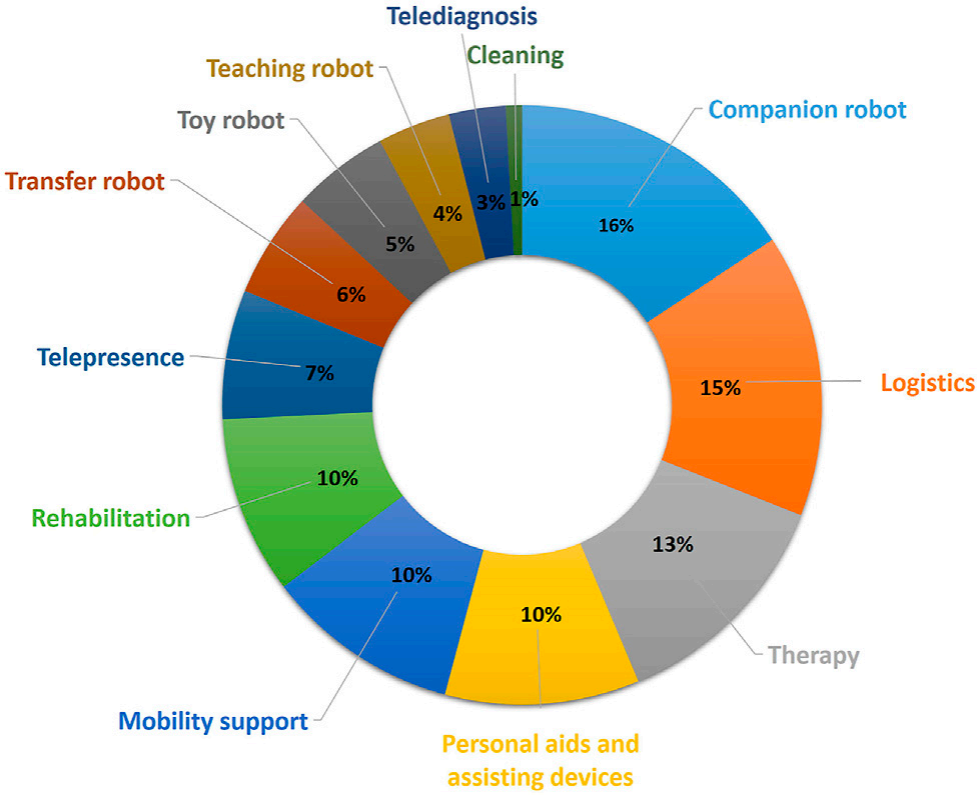
Percentage of project per each category of the use case classification.

Figure 10 shows the distribution of the projects along these classes. We can see that more than one-third of the projects are distributed across the three use cases: *Companion robots*, *Logistics*, and *Therapy*, the ones that are more focused on helping either caregiver or patient and simplifying their tasks. There are correspondingly fewer projects in the other use cases.

Some robots can be used in multiple scenarios. They can, for example, be used in hospitals, care centers, at home, etc., and they can also be used to provide support to different people, for example, to the patient as well as their caregivers or relatives. Therefore, a single robot or project may support more than one use case.

### 3.3 Technical vs. Use Case

In the previous sections, we have analyzed both the technical characteristics and use case scenarios. However, the relationship between these two classifications is also of interest. Table 1 shows how many projects from each technical category support which use case category. The empty cells indicate that there are no projects categorized in that intersection.

**TABLE 1:** Technical vs. use case classification.

			Technical classification						
			Wearable robotics	Teleoperated devices	Cobotic	Responsive	Cooperative	Collaborative	Supportive
Use case classification	Peripheral activities of nursing	Logistics		1			4	9	**23**
		Transfer robot	1				10		2
		Cleaning						1	1
	Increase autonomy of patient	Companion robot			1	4	2		**29**
		Personal aids	1	1	4			10	9
		Mobility support	7		1		2	1	**13**
		Therapy	1	1		7	5	1	14
		Toy robots		1		1	3	2	5
	Close involvement of patient and nurse	Rehabilitation	9				**11**	1	1
		Teaching robots				1	6	1	1
	Tele applications	Telepresence		2		2	2	1	10
		Telediagnosis	2	2				1	2

Before analyzing the relationship between these two categories, it is important to clarify that a project could have more than one use case scenario, as the robot capabilities could be used to perform different tasks that are not always covered by only one of them. However, the majority of systems have only one technical category, as the technical characteristics of the robot generally do not change. Although, in some cases, where the robot’s degree of autonomy can change, it is possible to find more than one technical category. For this reason, it is possible to find in the analysis examples of robots that can be assigned to more than one class of the use case classification. This explains why the sum of all cells in Table 1, 263, is significantly higher than the number of projects reported in Section 3, 133.

The highest concentration of projects is found in the last three columns, that is, spread across the *Cooperative*, *Collaborative*, and *Supportive* categories of the technical classification. This means that in each of these categories, it is possible to find a robot for the majority of use cases. A plausible explanation of the high presence of projects in these categories is the fact that the settings where they are developed are directly related with the activities of daily living (ADL), scenarios where service robots have been already introduced in order to help with these activities. The advancement of the technology allows their evolution to more care-related scenarios.

We can conclude that for the use cases of logistics, companion robots, and mobility support, there is a greater offer of projects or products within the *supportive* category. For the first two use cases, we can assume it is because they present situations where the robot and the human do not have to share a task. In fact, the robot performs a task for the user, e.g., bringing objects from another place (logistics), reducing the feeling of loneliness by playing entertaining multimedia for the user, reminding them to take their medicines, or performing a physical activity and keeping in touch with relatives by means of video calls (companion robot). In the case of the mobility support, the interaction is from a closer distance with the patient, as it might assist the user with the standing up and sitting down. But it can also perform actions from a longer distance and without interfering with them, such as monitoring the movement of the patient and informing some caregiver or relative in case of a fall; for this reason, this type of use case is also very related with the supportive category. There is an interesting intersection worth further exploration between logistics and teleoperated devices. This kind of robot can be used to perform logistics actions in environments where it is not safe for the nurse to go, for example, in quarantine areas, and in this way, the nurse can control the robot and still perform the actions reducing the risk of infection.

In the case of rehabilitation, the biggest offer of robots is found in the *cooperative* class. In this category, the robot and the user work together to fulfill a task, keeping a direct or an indirect contact. That is why they are so useful in rehabilitation activities, where the caregiver can show the exercise to the robot once, and it can repeat it exactly as many times as needed with the patient. With 9 projects versus 11, *wearable robotics* is also an option for the rehabilitation use case. Most of these projects are exoskeletons that are attached to the patient’s body and assist in the movements and improve the rehabilitation effectiveness. The closeness to the cooperative category indicates that it can be an interesting field for further exploration and development.

For the class of personal aids, there are robots available from the categories *collaborative*, where robots can help the user perform an activity of daily life, for example, eating or brushing their teeth. *Supportive* robots can provide information to the user that they might need for the day, like upcoming events, or they can assist bringing objects that are out of reach. For the class cobotic, the number of projects is shorter. These robots have less autonomy, so the assistance that they can provide on daily life activities is smaller, but they can be used to help the user organize the day and remind them of important dates or to take medication. Lastly, only one project can be found in the categories *wearable robotics* and *teleoperated devices*. In the first case, the sensors attached to the user’s body and connected with the robot can provide information that allows the robot to assist the person, for example, indicating to them that it is time to move, or by calling someone if it detects an abnormal situation, like a fall. The control of a teleoperated device can allow a relative or the caregiver to help the user perform activities of daily life, for example, a grandmother could cook with her grandchildren if they through the robot perform activities like cutting. Because of the small amount of projects available in these classes, *cobotic*, *wearable robotics*, and *teleoperated devices* projects for personal aids could also be an interesting investigation field.

In the category therapy, we find that the two classes with bigger amount of projects are *supportive* and *responsive*. In the first case, we can deduce that this may be due to the fact that most of the robots are home care robots which execute exercises to develop the cognitive skills of the user. That means they operate very independently and need no supervision from caregivers. In the case of *responsive* robots, we can deduce that these are the ones used in therapies, where the objective is to reduce the isolation of the patient, so the interaction between the robot and the user is closer. The amount of projects in the categories *cooperative* and *collaborative* are lower, but these classes could be a potential field of interest for those therapies where the caregiver needs the assistance of the robot or to share tasks, like when working with children with autism.

The *cooperative* category holds the largest number of projects for the transfer robot use case. It is possible to reason that this is because in this class, the robot acts as an independent agent that shares control of the task with the nurse. In this case, the mobilization of the patient always requires a nurse to ensure that the patient is well during the process and, at the same time, such a robot can significantly relieve the caregivers from the physical effort of the task. *Wearable robotics* and *supportive* categories have one and two projects, respectively, for the transfer of patients, which shows that they can also be potential fields for further investigation.

Lastly, the fact that the majority of projects in the telepresence category is *supportive* is not expected, but explainable. As we have pointed out, one robot can be used for more than one use case scenario, and telepresence robots are also used as companion and therapy robots. In both cases, these robots have a great deal of autonomy, but in certain situations, the caregiver or family member can control them. This explains that this use case has a majority of *supportive* projects, even though it is a scenario with low autonomy.

As stated in the above paragraph, the category *supportive* robots holds the highest amount of the studied projects. This can be explained due to the high presence of this kind of robots in the industry, the field where robots were first introduced. As the technology evolved, they were introduced in other fields, like service robotics, where they perform a role in households. This kind of robot looks to assist the person in the home environment, which led to the use of these robots in nursing care at home. The main use cases for nursing at home are in the category *Systems that increase the autonomy of the patient*, which explains the big cluster identified in Figure 9.

## 4 Discussion

The results of this review show research projects that are being developed worldwide, while providing an analysis of the most interesting fields and possible gaps for future research and commercial developments. The results identify as well which of these projects are already available as a product to be used in care. Finally, the review also helps to determine the different use case scenarios where robots can assist and present their technical characteristics.

We analyzed a total of 133 projects and classified them according to a technical point of view, extending the work of Haddadin and Croft (2016), and according to a use case classification defined by the authors. Because of the triangle established among the robot, patient, and nurse, we introduced a new systematic scheme for robots in nursing care, which allows for a clear assignment of the classes defined in the other two classification schemes in the new set categories.

When we compare the definitions of nursing care and the robotic systems, we can see that they cover several of the aspects of these definitions.

One of the main roles of the nurse in care is to act as an assistant of the patient. When the robot enters this interaction, a collaboration between the three of them is required. For this reason, the projects where a collaborative activity is developed are the majority.

As Figure 9 shows, a big part of the projects belong to the category *Systems that increase autonomy of the patient*, which means, many projects help the patient to live more independently, one of the main objectives of nursing care according to early and modern definitions. We can explain the high presence of projects in this category because it is a setting for the typical use of service robots, which serve as a basis for the development of these new, more care-oriented projects. Nevertheless, projects inside this category have barely any or no interaction with the nurse, which means that the nurse cannot control them. This fact contradicts the definition of nursing care, which states that the nurse is the one that initiates and controls the action. Therefore, a gap is identified here for further research on including the nurse in such systems.

Our data show that most robotic systems in this review did not replace nurses but exhibited the triangle introduced in this article. Thus, they fulfill both definitions of nursing in this regard, which emphasize the importance of interpersonal interaction.

One of the main contributions of this work is the classification of the projects into two different schemes. First, a technical classification is extended from Haddadin and Croft (2016) to consider the interaction with both patient and nurse, and second, a novel data-derived hierarchical use case classification is developed based on the analysis of the projects. The four main categories of this second classification are deduced from the technical classification, after the consideration of the triangle formed by the patient, nurse, and robot, and as we have shown in Figure 9, these four classes are well defined, so that the projects can be clearly assigned.

After evaluating the projects, we can find a correlation between these four classes and the phases of nursing care. In the early stages of care after admission, the patient needs a higher assistance from the nurse, and here, we can find the strongest representation of the triangle relationship explained along this work. In this first phase, we can identify those use cases belonging to the *Systems that closely involve both patient and nurse* category, such as rehabilitation and teaching robots. As the recovery advances, the patient gains independence and the nurse is required less, which means that projects inside the category *Systems that increase autonomy of the patient*, such as *Companion robots*, for *mobility support* or *assisting devices*, are more present in this stage. The class *Telemanipulation* stays in an intermediate stage, where the patient has more independence but still needs assistance from the nurse, however, this assistance can be done remotely, saving (travel) time and energy for the care professional. Lastly, the category *Peripheral activities of nursing* that includes activities like logistics or transport of patients covers the activities that are not at the core of nursing care, but may occur at any point during any phase.

As we have shown, there are plenty of research projects being developed in the field of robotics for care. This means that they are at an early stage and not yet ready for the market. Nevertheless, some of these projects might become products in a few years. Although, a big part of these will stay as research projects, and they will not get a medical device certification. A similar situation in the medical robotics sector reinforces this prediction. Hereto, there have been many research projects concerning robotic systems for quite some time, but only a few exceptions, like the *da Vinci* robots (Kim et al., 2002), have made it into clinical practice.

Because of the increase in the aging population, the need for care is a problem that needs to be addressed broadly. Taking Germany as an example and based on the study made in German Federal Ministry of Education and Research (2019b), we can see that the number of people in need of care will double (from 2.8 million to 4.6 million) in 40 years, while the number of caregivers will decrease. This humanitarian challenge creates an urgent need to develop the kind of technology defined in the different robotic projects studied in this work as well as within the identified gaps.

One topic that is of great importance for robotic systems in nursing care, which has not been discussed in this article, is the safety of human–robot interaction because robots often work in close proximity to patients, nurses, or both. A survey of this topic is provided by Zacharaki et al. (2020).
