# Supplementary material for: A Survey of Robotic Systems for Nursing Care
Source: Front Robot AI. 2022 Apr 7;9:832248. doi: 10.3389/frobt.2022.832248 (PMC9021873; doi:10.3389/frobt.2022.832248)
Supplement: Supplementary file 1 [file DataSheet1.docx]

Supplementary Material

# Supplementary Data

Supplementary Table 1 presents the list of all the found projects, along with their origin country and references.

## Technical classification of robotic technologies in Care

As we explained in the main text, the first classification used was a technical one, taken from (Haddadin and Croft, 2016) and based on two parameters: proximity, which refers to the closeness between the user and the robot and, in agency, which points out how independent the robot is, that is, what percentage of the robot actions are directly controlled by the human. The seven categories are explained in more detail in the following paragraphs.

- **Wearable robotics:** This type of robots are wearable devices, designed to be worn by humans (either nurses or patients). Therefore, they have to have an adaptable shape and fulfill kinematic and weight criteria. Depending on their function, they can be classified into exoskeletons, if their objective is to augment and/or assist in a task and, into prosthetic robots, if their mission is to restore a human limb function (Frisoli, 2019). These robots have a low autonomy and are placed on the person's body.
- **Teleoperated devices:** This type of robots are remotely controlled. The human operator (nurse) receives information and feedback from the robot sensors and can control it from the distance. The autonomy of the robot is also low, but they are separated from the operator's body (but not the patient's), even though a part of them is still close enough, as they are controlled by the human by means of a remote control device, such as a joystick.
- **Cobotic and intelligent assist devices:** These robots are designed to collaborate with the human (nurses or patients) and to help them to perform daily life activities. These robots also are at hand's reach, but their autonomy is higher than in the previous class.
- **Responsive:** The interaction between this type of robots and the human (patient) is by means of touch. It has been shown that touch plays an important role in forming and maintaining social bonds, so these robots look for obtaining a behavioral, emotional and physiological response from the user (Willemse and van Erp, 2019). As the interaction is by means of touch, the proximity between the robot and the user is very close, these robots also present a high autonomy, since they have to react automatically to the user's actions.
- **Cooperative:** In this case the robots operate independently of the user (nurse or patient), instead of being a passive assistant. Human and robot share the control of the task in a continuous and cooperative way and work in direct or indirect physical contact, in the latter case by contact through a common object (Siciliano and Khatib, 2016). The robots operate independently of the user, so the autonomy of this type of robot is high and at the same time, the proximity is at hand's distance, as they operate in direct or indirect contact.
- **Collaborative:** These robots work with the human (nurse or patient) on a common task, in which the labor is divided between them. They have to complete the separated parts of the task that best suit their abilities (Siciliano and Khatib, 2016). Because of the separation of tasks, the distance between the robot and the user is bigger (arms length) and the independence is a little bit higher as for the previous categories.
- **Supportive:** This type of robot looks for the way of optimizing the human task's performance (nurses or patient) or objective, most of the times providing the human with the materials, information and tools to do so, but they are not at the center of the performance of the task (Siciliano and Khatib, 2016). These robots have the highest values, both in autonomy and proximity.

## Use case classification of robotic technologies in Care

Based on literature (Bulgheroni, 2016; of Robotics, 2016; Ben-Ari and Mondada, 2018) we defined an engineering-driving and actionable use case classification, which classes we explain shortly:

### Peripheral activities of nursing

- Logistics: Robots that perform logistics chores, such as transporting objects from one place to another
- Transport of patients / transfer robot: These are used to help the caregiver to lift and transport the patient.
- Cleaning: As the name indicates, these robots are used to perform cleaning chores.

### Systems that increase autonomy of the patient

- Companion robots: These robots seek to accompany the user and reduce feelings like isolation and loneliness.
- Personal aids and assisting devices: Used to help the user to carry out daily life activities.
- Mobility support: robots that help the patient to move. These robots can support movements like standing or sitting, as well as walking.
- Therapy support: Robots used to lead therapy sessions or to assist in them.
- Toy robots: They are used to distract and entertain the user, in order to reduce the feelings of isolation and loneliness. In most cases, they are also used to develop some skill by means of games.

### Systems that closely involve both patient and nurse

- Rehabilitation: Robots that help and assist in rehabilitation processes.
- Teaching robots: these robots aim at helping the user to develop a certain skill. It is common to find in this category robots to teach kids with autism social skills.

### Tele applications

- Telepresence: Remote controlled robots that allow the user to be somewhere else. This kind of robot is especially useful when trying to reduce the loneliness feeling in old users or to help caregivers and family members to monitor them.
- Diagnostic Systems / Telediagnosis: They allow the doctor to examine the patient and perform a diagnosis without being in the same room as the patient.

# Supplementary Figures and Tables

**Supplementary** **Table 1.** List of 113 projects identified in this review.

| **Project name** | **Country** | **References** |
| --- | --- | --- |
| Robot Suit Hal | Japan | (Cyberdyne, 2020) |
| Muscle Suit Care Assist | Japan | (Innophys, 2020) |
| ReStore | USA | (R. Robotics, 2020b) |
| ReWalk | USA | (R. Robotics, 2020a) |
| SASUKE | Japan | (Muscle, 2020) |
| RIBA | Japan | (Mukai et al., 2010; R.-T. C. C. for Human-Interactive Robot Research(RTC), 2020; Wilkinson, 2020) |
| PR2 | USA | (Erickson et al., 2018; Jason Maderer, 2018; IEEE, 2020) |
| ARNA | USA | (Dan Popa, 2017) |
| RI-MAN | Japan | (Robotics today, 2006b; Onishi et al., 2007) |
| RoNa | USA | (Hu et al., 2011; Ding et al., 2014; Mahrouche, 2015) |
| Core | Japan | (Robotics today, 2010c; Tokyo Skytree Town Campus, 2020) |
| AMOR | Spain | (Stoelen et al., 2016; C. I. U. M. RoboticsLab, 2020; ExactDynamics, 2020) |
| ASIBOT | Spain | (Giménez et al., 2006; Jardón et al., 2011; C. I. U. M. RoboticsLab, 2019) |
| KINOVA JACO | Canada, Germany and China | (KINOVA, 2020b) |
| OBI | Canada, Germany and China | (KINOVA, 2020a) |
| Baxter humanoid robot | United Kingdom | (Gao et al., 2015, 2016; Zhang et al., 2017, 2019; Personal Robotics Lab. Imperial College London, 2020) |
| ROBERT | Denmark | (Life Science robotics, 2020) |
| HelpMate | USA | (Evans et al., 1992; Krishnamurthy and Evans, 1992; Evans, 1994; NASA, 2003) |
| TUG | USA | (Mutlu and Forlizzi, 2008; Niechwiadowicz and Khan, 2008; Zhang et al., 2008; AETHON, 2020) |
| My Spoon | Japan | (Naotunna et al., 2015; SECOM, 2020) |
| iARM | Netherlands | (Assistive innovations, 2020) |
| PerMMA | USA | (Wang et al., 2012; University of Pittsburgh, 2020) |
| HANDY 1 | USA | (Topping, 1995; Mike Topping, 1998) |
| Care Robot Yurina | Japan | (Robotics today, 2009a; TechCrunch, 2010) |
| HelloSpoon | Mexico | (Robotics today, 2014) |
| Robotic Bed | Japan | (Robotics today, 2010g; Panasonic, 2020) |
| Toyota i-foot | Japan | (Bock et al., 2012; The Future of Things, 2020) |
| HUBO FX-1 | Korea | (Lee et al., 2006; Robotics today, 2007) |
| ReMeDi | Austria | (Stollnberger et al., 2014; Arent et al., 2016; C. for Human-Computer Interaction. Universität Salzburg, 2020) |
| Ava (Dr. Robot) | USA | (Robotics today, 2003; IEEE SPECTRUM, 2012) |
| MOXI | USA | (D. Robotics, 2020) |
| Pillo | USA | (Pillohealth, 2020) |
| Lio | Switzerland | (Bendel, 2020; F\&P Personal Robotics, 2020) |
| P-Care | Switzerland | (Bendel, 2018; Robotic und produktion, 2019) |
| HOSPI | Japan | (Panasonic, 2015, 2019; Robotics today, 2015a) |
| Keio Robot | Japan | (Robotics today, 2008; Takahashi et al., 2009) |
| Terapio | Japan | (Tasaki et al., 2015) |
| ATOM 7xp | USA | (Robotics today, 2010a; FutureBots, 2019) |
| Mario | Ireland | (European Commission, 2019c; Prof. Dympna Casey, 2019; eHealth Ireland, 2020) |
| Pepper | Europe | (Tanioka et al., 2019; Softbank Robotics, 2020b, 2020a) |
| Romeo | France | (Softbank Robotics, 2020c) |
| Hector | Europe | (E. Commission, 2012; Schroeter et al., 2013) |
| Maggie | Spain | (C. I. U. M. RoboticsLab, 2014) |
| HOBBIT | Vienna | (Mayer and Panek, 2013; Vincze et al., 2014; Hobbit, 2020) |
| Twendy-one | Japan | (TWENDY-ONE, 2007; Iwata and Sugano, 2009) |
| Giraff | Italien, Spain and Sweden | (Coradeschi et al., 2014; Barsocchi et al., 2016) |
| Texai | USA | (Willow garage, 2015; Robot Center, 2020) |
| Nancy | Singapore | (Ge et al., 2011a, 2011b) |
| JIBO | USA | (Rane et al., 2014; Robotics today, 2015b; Jibo, 2020) |
| Robovie | Japan | (Ishiguro et al., 2001; Robotics today, 2010h) |
| ROLA | Taiwan | (Song et al., 2008; Robotics today, 2009b) |
| Eva | Netherlands | (Delta Journalistic platform TU Delft, 2013; Robotics today, 2013; TUDelft, 2020) |
| Family Nanny Robot | China | (Robotics today, 2010d) |
| HuiHui | China | (Robotics today, 2010f) |
| Pearl | USA | (Pollack et al., 2002) |
| BUDDY | USA | (B. F. Robotics, 2020) |
| Zora/NAO | Europe | (Vital et al., 2013, 2018; Pulido et al., 2017; Zorabots, 2020) |
| Paro | Japan | (PARO Robots U.S., 2014) |
| Mini | Spain | (Salichs et al., 2016) |
| PLEO | China/USA | (Dimas et al., 2010; Pitsch and Koch, 2010; PLEOrb, 2012; Albo-Canals et al., 2015; Fernández-Baena et al., 2015) |
| Keepon | USA | (Kozima et al., 2004, 2005, 2009; BeatBots, 2020) |
| Zeno/Milo | China | (Hanson et al., 2009; Salvador et al., 2015; Hanson Robotics, 2020; Robtos.nu, 2020; Robtos4autism, 2020) |
| Vizzy | Portugal | (Moreno et al., 2016; Avelino et al., 2018; Institute for Systems and Robotics \| LISBOA, 2020) |
| Huggable | USA | (Robotics today, 2006a; Stiehl et al., 2006; Personal Robots Group, 2015; Jeong et al., 2018) |
| Taizo | Japan | (Matsusaka et al., 2009; Robotics today, 2009c) |
| Fujitsu Teddy Bear | Japan | (Robotics today, 2010e) |
| Kaspar | England | (Dautenhahn et al., 2009; Wainer et al., 2014a, 2014b; Huijnena et al., 2015; Wood et al., 2017; University of Hertfordshire, 2020) |
| Kibo | USA | (Albo-Canals et al., 2018; Tufts University, 2018; González-González et al., 2019) |
| Mero | Korea | (Lee et al., 2010; Robotics Today, 2010b) |
| Engkey | Korea | (Lee et al., 2010; Robotics Today, 2010a) |
| Robota Dolls | Switzerland | (Robotics Today, 1997; Billard et al., 1998; Dautenhahn and Billard, 2002) |
| RAMCIP | Greece | (Abdelnour et al., 2016; Peleka et al., 2018; European Commission, 2022b) |
| CYBERLEGs++ | Italy | (CYBERLEGs++; Martini et al., 2020; European Commission, 2021c) |
| CARESSES | Italy | (Bui et al., 2017; Dang et al., 2017; Sgorbissa et al., 2019; European Commission, 2020a) |
| ENDORSE | France | (Ramdani et al., 2019; ENDORSE, 2020; European Commission, 2021b) |
| ERXOS | Italy | (European Commission, 2016) |
| MoveCare | Italy | (Luperto et al., 2019b, 2019a; European Commission, 2020d; MoveCare, 2020) |
| GrowMeUp | Portugal | (European Commission, 2019b; Martins and Dias, 2020; Pal Robotics, 2022b) |
| EnrichMe | Italy | (Salatino et al., 2016; European Commission, 2020b; Pal Robotics, 2022a) |
| Florence | Netherlands | (Isken et al., 2011; Lowet et al., 2012; OFFIS, 2013; European Commission, 2019d) |
| SRS | United Kingdom | (Maple et al., 2012; European Commission, 2017b) |
| CARER-AID | United Kingdom | (Conti et al., 2018, 2020; European Commission, 2019a) |
| ICone | Italy | (European Commission, 2020c; Heaxel, 2021) |
| SPEXOR | Slovenia | (Spexor, 2016; Chiara et al., 2017; European Commission, 2020e) |
| SocialRobot | Portugal | (European Commission, 2017c; Portugal et al., 2019) |
| MARSI | Spain | (Puyuelo-Quintana et al., 2020; European Commission, 2022a; Marsi-Bionics, 2022) |
| TRINA | USA | (Li et al., 2017; Worcester Polytechnic Institute, 2022) |
| SMiLE | Germany | (DLR: German Aeroespace Center, 2018; DLR: Institute of Robotics and Mechatronics, 2022) |
| ReHyb | Germany | (Pilla et al., 2020; European Commission, 2021a; ReHyb, 2022) |
| IWARD | Germany | (European Commission, 2012; APT, 2022) |
| ALFRED | Germany | (Josué et al., 2015; ALFRED, 2016; European Commission, 2017a) |
| PowerGraps | Germany | (Design Research Lab, 2018; German Federal Ministry of Education and Research, 2018a; Kuschan et al., 2018) |
| Recupera REHA | Germany | (Deutsches Forschungszentrum für Künstliche Intelligenz GmbH, 2020) |
| CareJack | Germany | (German Federal Ministry of Education and Research, 2015a; Klinikum Stadt Soest, 2016; Kostelnik, 2016; Kuschan et al., 2016, 2017; Moritz, Christoph and Hahn, 2016; Mularczyk, 2016; OTW Orthopädietechnik Winkler, 2016; Wolschke, Mirko and Liebach, Jana and Sommerfeld, Kamila and Smurawski, 2016) |
| MOBOT | Germany | (MOBOT, 2013; Fotinea et al., 2015; Efthimiou et al., 2016b, 2016a) |
| SCITOS G5 | Germany | (Müller et al., 2007; Duckett et al., 2013; MetraLabs mobile robots, 2020) |
| AuRoRoll | Germany | (German Federal Ministry of Education and Research, 2017; Wimmer, Christian and Urquizar, Carlos and Hammer, Mikael and Saez, 2017) |
| Rhoni | Germany | (Robotics Today, 2008; Buxbaum and Sen, 2018; Buxbaum et al., 2019) |
| SeRoDi | Germany | (Fraunhofer IPA, 2020g, 2020f; Hülsken-Giesler and Remmers, 2020; SeRoDi. Servicerobotik in der Pflege, 2020) |
| MIT-engAge | Germany | (Reichel et al., 2017; German Federal Ministry of Education and Research, 2020i; MTI-engage, 2020) |
| WiMi-Care | Germany | (Jacobs and Graf, 2012; Universität Duisburg-Essen, 2014; Fraunhofer IPA, 2020h) |
| MobIPaR | Germany | (Kersten and Brukamp, 2018; Servaty et al., 2018; German Federal Ministry of Education and Research, 2020f; MobIPaR-Projekt, 2020) |
| Elevon | Germany | (Fraunhofer IPA, 2020d) |
| SafeAssistance | Germany | (German Federal Ministry of Education and Research, 2016b; Heiligensetzer, Peter and Ott, 2016; Reif, Wolfgang and Hoffmann, 2016; Rosenberg, 2016) |
| Care-O-Bot 4 | Germany | (Schaeffer and May, 1999; Kittmann et al., 2015; Fraunhofer IPA, 2020c) |
| Care-O-Bot 3 | Germany | (Graf et al., 2009; Robotics today, 2010b; Fraunhofer IPA, 2020b) |
| ROREAS | Germany | (German Federal Ministry of Education and Research, 2016a; Gross et al., 2017b, 2017a; Roreas, 2017) |
| ALIAS | Germany | (Goetze et al., 2012; Ihsen et al., 2012; Mayer et al., 2012; Geiger et al., 2014a, 2014b; Rehrl et al., 2014; AAL PROGRAMME, 2020; Hülsken-Giesler and Remmers, 2020) |
| SYMPARTNER | Germany | (German Federal Ministry of Education and Research, 2018c; SYMPARTNER, 2018; Gross et al., 2019; Löffler et al., 2020) |
| OurPuppet | Germany | (Kuhlmann et al., 2018; Naroska et al., 2018; German Federal Ministry of Education and Research, 2019; OurPuppet, 2019) |
| ASARob | Germany | (ASARob, 2020; Fraunhofer IPA, 2020a; German Federal Ministry of Education and Research, 2020c) |
| RoPHa | Germany | (Fraunhofer IPA, 2020e; German Federal Ministry of Education and Research, 2020p; RoPHa, 2020) |
| KoBo34 | Germany | (Jahn et al., 2019; German Federal Ministry of Education and Research, 2020e; Technische Hochschule Rosenheim, 2020) |
| RobotKoop | Germany | (German Federal Ministry of Education and Research, 2020n; Hochschule Ravensburg-weingarten, 2020; RobotKoop, 2020) |
| MAID | Germany | (German Federal Ministry of Education and Research, 2015b; Guhl, 2015; Irgenfried, Stephan and Schneider, 2016; Wagner, 2016) |
| EmoRobot | Germany | (Hochschule Bonn-Rhein-Sieg, 2016; Prassler, Erwin and Ziegler, Sven and Bleses, Helma M. and Füller, 2017) |
| ROBINA | Germany | (Zeilfelder et al., 2018; Busch et al., 2019; German Federal Ministry of Education and Research, 2020m; ROBINA, 2020) |
| Reeti | Germany | (ROBOTSVOICE, 2013; Meschtscherjakov et al., 2016; Ritschel and André, 2017; Ritschel et al., 2018; Reeti, 2020; RobotShop, 2020; Universität Augsburg, 2020) |
| MOPASS | Germany | (Kuzmicheva et al., 2015, 2016; Universität Bremen, 2015; Martinez et al., 2016) |
| RoSylerNT | Germany | (MEDICA Magazine, 2019; German Federal Ministry of Education and Research, 2020q; KIT. Karlsruhe Institute of Technology, 2020) |
| REHATHESE | Germany | (Bastian, 2018; Blab, Florian and Starker, Felix and Czapka, Philip and Rogge, 2018; German Federal Ministry of Education and Research, 2018b; Keiser, Thomas and Hoppe, 2018; Larsson, 2018; Müller, 2018) |
| ERimAlter | Germany | (Goethe Universität, 2014; Kolling, Thorsten and Klein, Barbara and Knopf, Monika and Oswald, Frank and Pantel, 2015; Baisch et al., 2017) |
| SE_BURG | Germany | (German Federal Ministry of Education and Research, 2016c; Hacker, Steffen and Dürselen, 2017; Ivlev, Oleg and Wilkening, André and Stöppler, Henning and Baiden, David and Wang, Chen and Xu, Chunya and Weishaupt, Mark and Sorgenicht, 2017; Koch, Christian and Haas, Hans-Dieter and Henni-Mansour, Karim and Fischer, 2017; Krischak, Gert and Knapp, Sebastian and Knoll, 2017; Resinger, 2017) |
| AdaMeKoR | Germany | (German Federal Ministry of Education and Research, 2020a) |
| ArNe | Germany | (German Federal Ministry of Education and Research, 2020b) |
| HoLLiECares | Germany | (German Federal Ministry of Education and Research, 2020d) |
| MobiStaR | Germany | (German Federal Ministry of Education and Research, 2020g) |
| Morphia | Germany | (German Federal Ministry of Education and Research, 2020h; MORPHIA, 2020) |
| PeTRA | Germany | (German Federal Ministry of Education and Research, 2020j; Hochschule Karlsruhe Technik und Wirtschaft, 2020) |
| PfleKoRo | Germany | (AME \| RWTHAACHEN UNIVERSITY, 2019; German Federal Ministry of Education and Research, 2020k) |
| REsPonSe | Germany | (German Federal Ministry of Education and Research, 2020l) |
| RoMi | Germany | (German Federal Ministry of Education and Research, 2020o) |
| RUBY Demenz | Germany | (German Federal Ministry of Education and Research, 2020r) |

# References

AAL PROGRAMME (2020). ALIAS. Available at: http://www.aal-europe.eu/projects/alias/ [Accessed December 7, 2021].

Abdelnour, C., TantinyaNatalia, Hernandez, J., Martin, E., Garcia, S., Ribes, J., et al. (2016). RAMCIP PROJECT , A ROBOTIC ASSISTANT TO SUPPORT ALZHEIMER ’ S DISEASE PATIENTS AT HOME : A NOVEL APPROACH IN CAREGIVING. *Alzheimer’s \& Dement.* 12, P798--P798. doi:10.1016/j.jalz.2016.06.1610.

AETHON (2020). TUG. Change Healthcare. Available at: https://aethon.com/mobile-robots-for-healthcare/ [Accessed December 8, 2021].

Albo-Canals, J., Fernández-Baena, A., Boldu, R., Barco, A., Navarro, J., Miralles, D., et al. (2015). Enhancing long-term children to robot interaction engagement through cloud connectivity. in *Proceedings of the Tenth Annual ACM/IEEE International Conference on Human-Robot Interaction Extended Abstracts*, 105–106.

Albo-Canals, J., Martelo, A. B., Relkin, E., Hannon, D., Heerink, M., Heinemann, M., et al. (2018). A pilot study of the KIBO robot in children with severe ASD. *Int. J. Soc. Robot.* 10, 371–383.

ALFRED (2016). Interactive Assistant for Independent Living and Active Ageing. Available at: https://alfred.eu/index.html [Accessed January 19, 2022].

AME | RWTHAACHEN UNIVERSITY (2019). PfleKoRo: Erleichterte Pflege schwer zu pflegender Patienten durch Kooperierende Robotik. Available at: https://www.ame.rwth-aachen.de/cms/AME/Forschung/RPE-Rehabilitations-und-Praeventionst/Rehabilitation/~donpt/PfleKoRo/ [Accessed December 8, 2021].

APT (2022). Intelligent Robot Swarm for Attendance, Recognition, Cleaning and Delivery (IWARD). Available at: https://aptcentre.ie/projects/intelligent-robot-swarm-for-attendance-recognition-cleaning-and-delivery-iward/ [Accessed January 14, 2022].

Arent, K., Jakubiak, J., Drwi\kega Michałand Cholewiński, M., Stollnberger, G., Giuliani, M., Tscheligi, M., et al. (2016). Control of mobile robot for remote medical examination: Design concepts and users’ feedback from experimental studies. in *2016 9th International Conference on Human System Interactions (HSI)*, 76–82.

ASARob (2020). ASARob. Aufmerksamkeits-Sensitiver AssistenzRoboter. Available at: https://asarob.de/ [Accessed June 11, 2020].

Assistive innovations (2020). iARM. Available at: https://www.assistive-innovations.com/robotic-arms/iarm [Accessed December 8, 2021].

Avelino, J., Simão, H., Ribeiro, R., Moreno, P., Figueiredo, R., Duarte, N., et al. (2018). Experiments with vizzy as a coach for elderly exercise. in *Proc. Workshop Pers. Robots Exercising Coaching-HRI Conf.(PREC)*, 1–6.

B. F. Robotics (2020). BUDDY. The emotional robot. Available at: https://buddytherobot.com/en/buddy-the-emotional-robot/ [Accessed December 7, 2021].

Baisch, S., Kolling, T., Schall, A., Rühl, S., Selic, S., Kim, Z., et al. (2017). Acceptance of social robots by elder people: does psychosocial functioning matter? *Int. J. Soc. Robot.* 9, 293–307.

Barsocchi, P., Cesta, A., Coraci, L., Cortellessa, G., De Benedictis, R., Fracasso, F., et al. (2016). The giraffplus experience: from laboratory settings to test sites robustness (short paper). in *2016 5th IEEE international conference on cloud networking (Cloudnet)*, 192–195.

Bastian, R. (2018). Verbundprojekt “Orthopädische Rehabilitation der unteren Extremitäten durch aktive Orthesen - REHATHESE”, Teilprojekt “Neues Leichtbaukonzept und Gesamtintegration” : Abschlussbericht : Laufzeit: 01.04.2015-31.05.2018, REHATHESE, Orthopädische Rehabil. Available at: https://www.tib.eu/de/suchen/id/TIBKAT%3A1049141091 [Accessed December 8, 2021].

BeatBots (2020). My Keepon. Available at: https://beatbots.net/my-keepon [Accessed December 8, 2021].

Ben-Ari, M., and Mondada, F. (2018). “Robots and Their Applications,” in *Elements of Robotics* (Cham: Springer International Publishing), 1–20. doi:10.1007/978-3-319-62533-1_1.

Bendel, O. (2018). “Pflegeroboter,” in (Springer Nature), 37–58.

Bendel, O. (2020). Co-robots as care robots. *arXiv Prepr. arXiv2004.04374*.

Billard, A., Dautenhahn, K., and Hayes, G. (1998). Experiments on human-robot communication with Robota, an imitative learning and communicating doll robot. in *Proc. Socially Situated Intelligence Workshop*.

Blab, Florian and Starker, Felix and Czapka, Philip and Rogge, T. (2018). KMU-innovativ - Verbundprojekt: “REHATHESE”: Abschlussbericht 2018, REHATHESE. doi:10.2314/KXP:1667308645.

Bock, T., Linner, T., and Ikeda, W. (2012). Exoskeleton and humanoid robotic technology in construction and built environment. *Futur. Humanoid Robot. Appl.*, 111–144.

Bui, H. D., Pham, C., Lim, Y., Tan, Y., and Chong, N. Y. (2017). Integrating a Humanoid Robot into ECHONET-Based Smart Home Environments. *Lect. Notes Comput. Sci. (including Subser. Lect. Notes Artif. Intell. Lect. Notes Bioinformatics)* 10652 LNAI, 314–323. doi:10.1007/978-3-319-70022-9_31.

Bulgheroni, M. (2016). Analysis of existing robots classifications. *Ab.Acus. [Internal Reel. Proj. Doc.*

Busch, T., Zeilfelder, J., Zhou, K., and Stork, W. (2019). A jaw based human-machine interface with machine learning. in *2019 IEEE Sensors Applications Symposium (SAS)*, 1–6.

Buxbaum, H., and Sen, S. (2018). “Kollaborierende Roboter in der Pflege--Sicherheit in der Mensch-Maschine-Schnittstelle,” in *Pflegeroboter* (Springer Gabler, Wiesbaden), 1–22.

Buxbaum, H., Sen, S., and Kremer, L. (2019). An Investigation into the Implication of Human-Robot Collaboration in the Health Care Sector. *IFAC-PapersOnLine* 52, 217–222.

C. for Human-Computer Interaction. Universität Salzburg (2020). ReMeDi- Remote Medical Diagnostician. Available at: https://hci.sbg.ac.at/remedi/ [Accessed December 7, 2021].

C. I. U. M. RoboticsLab (2014). Maggie. Available at: http://roboticslab.uc3m.es/roboticslab/robot/maggie [Accessed December 7, 2021].

C. I. U. M. RoboticsLab (2019). ASIBOT. Available at: http://roboticslab.uc3m.es/roboticslab/robot/asibot [Accessed December 7, 2021].

C. I. U. M. RoboticsLab (2020). AMOR. Available at: http://roboticslab.uc3m.es/roboticslab/robot/amor [Accessed December 8, 2021].

Chiara, M., Silvestro, C., Jos, M., Symposium, I., and Robotics, W. (2017). Wearable Robotics: Challenges and Trends. 16, 311–315. doi:10.1007/978-3-319-46532-6.

Conti, D., Trubia, G., Buono, S., Di Nuovo, S., and Di Nuovo, A. (2018). *Evaluation of a robot-assisted therapy for children with autism and intellectual disability*. Springer International Publishing doi:10.1007/978-3-319-96728-8_34.

Conti, D., Trubia, G., Buono, S., Di Nuovo, S., and Di Nuovo, A. (2020). Social robots to support practitioners in the education and clinical care of children: The CARER-AID project. *Life Span Disabil.* 23, 17–30.

Coradeschi, S., Cesta, A., Cortellessa, G., Coraci, L., Galindo, C., Gonzalez, J., et al. (2014). “GiraffPlus: a system for monitoring activities and physiological parameters and promoting social interaction for elderly,” in *Human-Computer Systems Interaction: Backgrounds and Applications 3* (Springer), 261–271.

Cyberdyne (2020). What’s HAL? Available at: https://www.cyberdyne.jp/english/products/HAL/index.html [Accessed December 7, 2021].

CYBERLEGs++ The CYBERnetic LowEr-Limb CoGnitive Ortho-prosthesis Plus Plus. Available at: http://www.cyberlegs.eu/ [Accessed January 13, 2022].

D. Robotics (2020). Meet MOXI. Available at: https://diligentrobots.com/ [Accessed December 7, 2021].

Dan Popa (2017). PFI:BIC - Adaptive Robotic Nursing Assistants for Physical Tasks in Hospital Environments. Available at: https://www.nsf.gov/awardsearch/showAward?AWD_ID=1643989 [Accessed December 7, 2021].

Dang, T. L. Q., Jeong, S., and Chong, N. Y. (2017). Personalized robot emotion representation through retrieval of memories. *2017 3rd Int. Conf. Control. Autom. Robot. ICCAR 2017*, 65–70. doi:10.1109/ICCAR.2017.7942662.

Dautenhahn, K., and Billard, A. (2002). “Games children with autism can play with Robota, a humanoid robotic doll,” in *Universal access and assistive technology* (Springer), 179–190.

Dautenhahn, K., Nehaniv, C. L., Walters, M. L., Robins, B., Kose-Bagci, H., Assif, N., et al. (2009). KASPAR--a minimally expressive humanoid robot for human--robot interaction research. *Appl. Bionics Biomech.* 6, 369–397.

Delta Journalistic platform TU Delft (2013). Handy robot serves drinks. Available at: https://www.delta.tudelft.nl/article/handy-robot-serves-drinks# [Accessed December 7, 2021].

Design Research Lab (2018). PowerGrasp – A Soft Robotic Assistive System. Available at: https://www.drlab.org/project/powergrasp-a-soft-robotic-assistive-system/ [Accessed December 8, 2021].

Deutsches Forschungszentrum für Künstliche Intelligenz GmbH (2020). Recupera REHA. Available at: https://robotik.dfki-bremen.de/de/forschung/projekte/recupera-reha.html [Accessed December 8, 2021].

Dimas, J., Leite, I., Pereira, A., Cuba, P., Prada, R., and Paiva, A. (2010). Pervasive pleo: long-term attachment with artificial pets. in *Mobile HCI*.

Ding, J., Lim, Y.-J., Solano, M., Shadle, K., Park, C., Lin, C., et al. (2014). Giving patients a lift-the robotic nursing assistant (RoNA). in *2014 IEEE International Conference on Technologies for Practical Robot Applications (TePRA)*, 1–5.

DLR: German Aeroespace Center (2018). Robots as care as­sis­tants for the el­der­ly – pre­sen­ta­tion of the SMiLE project. Available at: https://www.dlr.de/content/en/articles/news/2018/2/20180509_robots-as-care-assistants-for-the-elderly-presentation-of-the-smile-project_27306.html.

DLR: Institute of Robotics and Mechatronics (2022). SMiLE. Available at: https://www.dlr.de/rm/en/desktopdefault.aspx/tabid-12424/#gallery/29416.

Duckett, T., Hanheide, M., Krajnik, T., Pulido Fentanes, J., Dondrup, C., and others (2013). Spatio-temporal representation for cognitive control in long-term scenarios.

E. Commission (2012). Companionable research project delivers robotic assistance for the elderly. Available at: https://ec.europa.eu/digital-single-market/en/news/companionable-research-project-delivers-robotic-assistance-elderly [Accessed May 22, 2020].

Efthimiou, E., Fotinea, S.-E., Goulas, T., Dimou, A.-L., Koutsombogera, M., Pitsikalis, V., et al. (2016a). The MOBOT platform--showcasing multimodality in human-assistive robot interaction. in *International Conference on Universal Access in Human-Computer Interaction*, 382–391.

Efthimiou, E., Fotinea, S.-E., Goulas, T., Koutsombogera, M., Karioris, P., Vacalopoulou, A., et al. (2016b). The MOBOT rollator human-robot interaction model and user evaluation process. in *2016 IEEE Symposium Series on Computational Intelligence (SSCI)*, 1–8.

eHealth Ireland (2020). MARIO Managing active and healthy ageing using caring service robots. Available at: https://www.ehealthireland.ie/case studies/mario-managing-active-and-healthy-ageing-using-caring-service-robots/ [Accessed December 8, 2021].

ENDORSE (2020). ENDORSE PROJECT. Available at: http://www.endorse-project.eu/ [Accessed January 14, 2022].

Erickson, Z., Clever, H. M., Turk, G., Liu, C. K., and Kemp, C. C. (2018). Deep haptic model predictive control for robot-assisted dressing. in *2018 IEEE international conference on robotics and automation (ICRA)*, 4437–4444.

European Commission (2012). Intelligent robot swarm for attendance, recognition, cleaning and delivery. Available at: https://cordis.europa.eu/project/id/045254 [Accessed January 14, 2022].

European Commission (2016). ElectroRheological fluid based eXOSkeleton devices for physical upper limb rehabilitation. Available at: https://cordis.europa.eu/project/id/717644 [Accessed January 14, 2022].

European Commission (2017a). ALFRED - Personal Interactive Assistant for Independent Living and Active Ageing. Available at: https://cordis.europa.eu/project/id/611218 [Accessed January 19, 2022].

European Commission (2017b). Multi-Role Shadow Robotic System for Independent Living. Available at: https://cordis.europa.eu/project/id/247772 [Accessed January 17, 2022].

European Commission (2017c). SocialRobot. Available at: https://cordis.europa.eu/project/id/285870 [Accessed January 19, 2022].

European Commission (2019a). Controlled Autonomous Robot for Early detection and Rehabilitation of Autism and Intellectual Disability. Available at: https://cordis.europa.eu/project/id/703489 [Accessed January 17, 2022].

European Commission (2019b). GrowMeUp. Available at: https://cordis.europa.eu/project/id/643647 [Accessed January 14, 2022].

European Commission (2019c). Managing active and healthy aging with use of caring service robots. Available at: https://cordis.europa.eu/project/id/643808 [Accessed January 14, 2022].

European Commission (2019d). Multi Purpose Mobile Robot for Ambient Assisted Living. Available at: https://cordis.europa.eu/project/id/248730 [Accessed January 17, 2022].

European Commission (2020a). Culture Aware Robots and Environmental Sensor Systems for Elderly Support. Available at: https://cordis.europa.eu/project/id/737858 [Accessed January 14, 2022].

European Commission (2020b). Enabling Robot and assisted living environment for Independent Care and Health Monitoring of the Elderly. Available at: https://cordis.europa.eu/project/id/643691 [Accessed January 14, 2022].

European Commission (2020c). ICone: a novel device to scale up robotic rehabilitation and unlock the potential of motor recovery for stroke survivors. Available at: https://cordis.europa.eu/project/id/889555 [Accessed January 17, 2022].

European Commission (2020d). Multiple-actOrs Virtual Empathic CARgiver for the Elder. Available at: https://cordis.europa.eu/project/id/732158 [Accessed January 14, 2022].

European Commission (2020e). Spinal Exoskeletal Robot for Low Back Pain Prevention and Vocational Reintegration. Available at: https://cordis.europa.eu/project/id/687662 [Accessed January 17, 2022].

European Commission (2021a). Rehabilitation based on Hybrid neuroprosthesis. Available at: https://cordis.europa.eu/project/id/871767 [Accessed January 14, 2022].

European Commission (2021b). Safe, Efficient and Integrated Indoor Robotic Fleet for Logistic Applications in Healthcare and Commercial Spaces. Available at: https://cordis.europa.eu/project/id/823887 [Accessed January 14, 2022].

European Commission (2021c). The CYBERnetic LowEr-Limb CoGnitive Ortho-prosthesis Plus Plus. Available at: https://cordis.europa.eu/project/id/731931 [Accessed January 13, 2022].

European Commission (2022a). Disruptive technologies for effectively rehabilitating chronic ambulatory disability. Available at: https://cordis.europa.eu/project/id/784498 [Accessed January 19, 2022].

European Commission (2022b). Robotic Assistant for MCI patients at home. Available at: https://cordis.europa.eu/project/id/643433 [Accessed January 12, 2022].

Evans, J., Krishnamurthy, B., Barrows, B., Skewis, T., and Lumelsky, V. (1992). Handling real-world motion planning: a hospital transport robot. *IEEE Control Syst. Mag.* 12, 15–19.

Evans, J. M. (1994). HelpMate: An autonomous mobile robot courier for hospitals. in *Proceedings of IEEE/RSJ International Conference on Intelligent Robots and Systems (IROS’94)*, 1695–1700.

ExactDynamics (2020). AMOR. Available at: http://www.amorrobot.com/overview.shtml [Accessed December 8, 2021].

F\&P Personal Robotics (2020). Lio – Professional Personal Robot. Available at: https://www.fp-robotics.com/en/care-lio/ [Accessed December 8, 2021].

Fernández-Baena, A., Boldú, R., Albo-Canals, J., and Miralles, D. (2015). Interaction between Vleo and Pleo, a virtual social character and a social robot. in *2015 24th IEEE International Symposium on Robot and Human Interactive Communication (RO-MAN)*, 694–699.

Fotinea, S.-E., Efthimiou, E., Koutsombogera, M., Dimou, A.-L., Goulas, T., Maragos, P., et al. (2015). The MOBOT human-robot communication model. in *2015 6th IEEE International Conference on Cognitive Infocommunications (CogInfoCom)*, 201–206.

Fraunhofer IPA (2020a). ASARob: Aufmerksamkeitssensitiver AssistenzRoboter. Available at: https://www.ipa.fraunhofer.de/de/referenzprojekte/ASARoB.html [Accessed December 8, 2021].

Fraunhofer IPA (2020b). Care-O-bot 3. Available at: https://www.care-o-bot.de/en/care-o-bot-3.html [Accessed December 8, 2021].

Fraunhofer IPA (2020c). Care-O-bot 4. Available at: https://www.care-o-bot.de/en/care-o-bot-4.html [Accessed December 8, 2021].

Fraunhofer IPA (2020d). Elevon: Teilautonomer Lifter für die Aufnahme und den Transport von Personen. Available at: https://www.ipa.fraunhofer.de/de/referenzprojekte/Elevon.html [Accessed December 7, 2021].

Fraunhofer IPA (2020e). RoPHa: Robuste Perzeption für die interaktive Unterstützung älterer Nutzer bei Handhabungsaufgaben im häuslichen Umfeld. Available at: https://www.ipa.fraunhofer.de/de/referenzprojekte/RoPHa.html [Accessed December 7, 2021].

Fraunhofer IPA (2020f). SERVICEROBOTER-TECHNOLOGIEN FÜR DIE STATIONÄRE PFLEGE. Available at: https://www.ipa.fraunhofer.de/content/dam/ipa/de/documents/Kompetenzen/Roboter--und-Assistenzsysteme/300_434_Serviceroboter-Technologien für die stationäre Pflege.pdf [Accessed December 7, 2021].

Fraunhofer IPA (2020g). Serviceroboter in stationären Pflegeeinrichtungen. Available at: https://www.ipa.fraunhofer.de/content/dam/ipa/de/documents/Kompetenzen/Roboter--und-Assistenzsysteme/Serviceroboter_stationaereEinrichtungen.pdf [Accessed December 7, 2021].

Fraunhofer IPA (2020h). WiMi-Care: Förderung des Wissenstransfers für eine active Mitgestaltung des Pflegesektors durch Mikrosystemtechnik. Available at: https://www.ipa.fraunhofer.de/de/referenzprojekte/WiMi-Care.html [Accessed December 8, 2021].

Frisoli, A. (2019). “Wearable Robots,” in *Encyclopedia of Robotics*, eds. M. H. Ang, O. Khatib, and B. Siciliano (Berlin, Heidelberg: Springer Berlin Heidelberg), 1–8. doi:10.1007/978-3-642-41610-1_22-1.

FutureBots (2019). ATOM humanoid robot. Available at: http://www.futurebots.com/walk.htm [Accessed December 7, 2021].

Gao, Y., Chang, H. J., and Demiris, Y. (2015). User modelling for personalised dressing assistance by humanoid robots. in *2015 IEEE/RSJ International Conference on Intelligent Robots and Systems (IROS)*, 1840–1845.

Gao, Y., Chang, H. J., and Demiris, Y. (2016). Iterative path optimisation for personalised dressing assistance using vision and force information. in *2016 IEEE/RSJ international conference on intelligent robots and systems (IROS)*, 4398–4403.

Ge, S. S., Cabibihan, J.-J., Zhang, Z., Li, Y., Meng, C., He, H., et al. (2011a). Design and development of nancy, a social robot. in *2011 8th International Conference on Ubiquitous Robots and Ambient Intelligence (URAI)*, 568–573.

Ge, S. S., Safizadeh, M. R., and Li, Y. (2011b). Mechanical design of social robot Nancy. in *2011 IEEE/SICE International Symposium on System Integration (SII)*, 324–329.

Geiger, J., Leykauf, T., Rehrl, T., Wallhoff, F., and Rigoll, G. (2014a). “The robot ALIAS as a gaming platform for elderly persons,” in *Ambient Assisted Living* (Springer), 327–340.

Geiger, J., Yenin, I., Wallhoff, F., and Rigoll, G. (2014b). “Display of emotions with the robotic platform ALIAS,” in *Ambient Assisted Living* (Springer), 287–297.

German Federal Ministry of Education and Research (2015a). CareJack. Available at: https://www.interaktive-technologien.de/projekte/carejack [Accessed December 7, 2021].

German Federal Ministry of Education and Research (2015b). MAID. Available at: https://www.interaktive-technologien.de/projekte/maid [Accessed December 7, 2021].

German Federal Ministry of Education and Research (2016a). ROREAS. Available at: https://www.interaktive-technologien.de/projekte/roreas [Accessed December 8, 2021].

German Federal Ministry of Education and Research (2016b). SafeAssistance. Available at: https://www.interaktive-technologien.de/projekte/safeassistance [Accessed December 8, 2021].

German Federal Ministry of Education and Research (2016c). SE_BURG. Available at: https://www.interaktive-technologien.de/projekte/se_burg [Accessed December 8, 2021].

German Federal Ministry of Education and Research (2017). AuRoRoll. Available at: https://www.interaktive-technologien.de/projekte/auroroll [Accessed December 8, 2021].

German Federal Ministry of Education and Research (2018a). PowerGrasp. Available at: https://www.interaktive-technologien.de/projekte/powergrasp [Accessed December 7, 2021].

German Federal Ministry of Education and Research (2018b). REHATHESE. Available at: https://www.interaktive-technologien.de/projekte/rehathese [Accessed December 8, 2021].

German Federal Ministry of Education and Research (2018c). SYMPARTNER. Available at: https://www.interaktive-technologien.de/projekte/sympartner [Accessed December 7, 2021].

German Federal Ministry of Education and Research (2019). OurPuppet. Available at: https://www.interaktive-technologien.de/projekte/ourpuppet [Accessed December 7, 2021].

German Federal Ministry of Education and Research (2020a). AdaMekoR. Available at: https://www.interaktive-technologien.de/projekte/adamekor [Accessed December 7, 2021].

German Federal Ministry of Education and Research (2020b). ArNe. Available at: https://www.interaktive-technologien.de/projekte/arne [Accessed December 8, 2021].

German Federal Ministry of Education and Research (2020c). ASARob. Available at: https://www.interaktive-technologien.de/projekte/asarob [Accessed December 7, 2021].

German Federal Ministry of Education and Research (2020d). HoLLiECares. Available at: https://www.interaktive-technologien.de/projekte/holliecares [Accessed December 7, 2021].

German Federal Ministry of Education and Research (2020e). KoBo34. Available at: https://www.interaktive-technologien.de/projekte/kobo34 [Accessed December 7, 2021].

German Federal Ministry of Education and Research (2020f). MobIPaR. Available at: https://www.interaktive-technologien.de/projekte/mobipar [Accessed December 7, 2021].

German Federal Ministry of Education and Research (2020g). MobiStaR. Available at: https://www.interaktive-technologien.de/projekte/mobistar [Accessed December 8, 2021].

German Federal Ministry of Education and Research (2020h). MORPHIA. Available at: https://www.interaktive-technologien.de/projekte/morphia [Accessed December 8, 2021].

German Federal Ministry of Education and Research (2020i). MTI-engAge. Available at: https://www.interaktive-technologien.de/projekte/mti-engage [Accessed December 7, 2021].

German Federal Ministry of Education and Research (2020j). PeTRA. Available at: https://www.interaktive-technologien.de/projekte/petra [Accessed December 7, 2021].

German Federal Ministry of Education and Research (2020k). PfleKoRo. Available at: https://www.interaktive-technologien.de/projekte/pflekoro [Accessed December 7, 2021].

German Federal Ministry of Education and Research (2020l). REsPonSe. Available at: https://www.interaktive-technologien.de/projekte/response [Accessed December 8, 2021].

German Federal Ministry of Education and Research (2020m). ROBINA. Available at: https://www.interaktive-technologien.de/projekte/robina [Accessed December 8, 2021].

German Federal Ministry of Education and Research (2020n). RobotKoop. Available at: https://www.interaktive-technologien.de/projekte/robotkoop [Accessed December 8, 2021].

German Federal Ministry of Education and Research (2020o). RoMi. Available at: https://www.interaktive-technologien.de/projekte/romi [Accessed December 8, 2021].

German Federal Ministry of Education and Research (2020p). RoPHa. Available at: https://www.interaktive-technologien.de/projekte/ropha [Accessed December 8, 2021].

German Federal Ministry of Education and Research (2020q). RoSylerNT. Available at: https://www.interaktive-technologien.de/projekte/rosy_lernt [Accessed December 8, 2021].

German Federal Ministry of Education and Research (2020r). RUBYDemenz. Available at: https://www.interaktive-technologien.de/projekte/rubydemenz [Accessed December 8, 2021].

Giménez, A., Jardón, A., Correal, R., Cabas, R., and Balaguer, C. (2006). “A portable light-weight climbing robot for personal assistance applications,” in *Climbing and Walking Robots* (Springer), 961–968.

Goethe Universität (2014). Projekt ERimAlter. Available at: https://www.uni-frankfurt.de/53969262/ERimAlter [Accessed December 8, 2021].

Goetze, S., Fischer, S., Moritz, N., Appell, J.-E., and Wallhoff, F. (2012). Multimodal human-machine interaction for service robots in home-care environments. in *Proceedings of the 1st Workshop on Speech and Multimodal Interaction in Assistive Environments*, 1–7.

González-González, C. S., Herrera-González, E., Moreno-Ruiz, L., Reyes-Alonso, N., Hernández-Morales, S., Guzmán-Franco, M. D., et al. (2019). Computational thinking and down syndrome: An exploratory study using the KIBO robot. in *Informatics*, 25.

Graf, B., Reiser, U., Hägele, M., Mauz, K., and Klein, P. (2009). Robotic home assistant Care-O-bot®3-product vision and innovation platform. in *2009 IEEE Workshop on Advanced Robotics and its Social Impacts*, 139–144.

Gross, H.-M., Meyer, S., Scheidig, A., Eisenbach, M., Mueller, S., Trinh, T. Q., et al. (2017a). Mobile robot companion for walking training of stroke patients in clinical post-stroke rehabilitation. in *2017 IEEE International Conference on Robotics and Automation (ICRA)*, 1028–1035.

Gross, H.-M., Scheidig, A., Debes, K., Einhorn, E., Eisenbach, M., Mueller, S., et al. (2017b). ROREAS: robot coach for walking and orientation training in clinical post-stroke rehabilitation—prototype implementation and evaluation in field trials. *Auton. Robots* 41, 679–698.

Gross, H.-M., Scheidig, A., Müller, S., Schütz, B., Fricke, C., and Meyer, S. (2019). Living with a mobile companion robot in your own apartment-final implementation and results of a 20-weeks field study with 20 seniors. in *2019 International Conference on Robotics and Automation (ICRA)*, 2253–2259.

Guhl, T. (2015). MAID - Mobilitätsassistent zur Unterstützung bewegungseingeschränkter Personen : Abschlussbericht zum Verbundvorhaben : Teilvorhaben: Entwicklung der Hardwareplattform inklusive autonomer Navigation. doi:10.2314/GBV:874383838.

Hacker, Steffen and Dürselen, L. (2017). Verbundprojekt: Intuitiv bedienbare bewegungsunterstützende Robotergeräte mit Selbstanpassung an den Nutzer; Teilvorhaben: Biomechanische Aspekte : BMBF-Verbundprojekt SE\_BURG : Abschlussbericht : Laufzeit des Vorhabens: 01.01.2014-31.12.2016, Final repo. Available at: https://www.tib.eu/de/suchen/id/TIBKAT%3A894421212 [Accessed December 8, 2021].

Haddadin, S., and Croft, E. (2016). “Physical human--robot interaction,” in *Springer handbook of robotics* (Springer), 1835–1874.

Hanson, D., Baurmann, S., Riccio, T., Margolin, R., Dockins, T., Tavares, M., et al. (2009). Zeno: A cognitive character. in *Ai magazine, and special proc. of aaai national conference, chicago*.

Hanson Robotics (2020). Zeno. Available at: https://www.hansonrobotics.com/zeno/ [Accessed December 8, 2021].

Heaxel (2021). icone® Smart Rehabilitation System. Available at: https://heaxel.com/products/ [Accessed January 17, 2022].

Heiligensetzer, Peter and Ott, M. (2016). SafeAssistance : intelligente Hinderniserkennung mit kapazitiven Sensoren zur sicheren Mensch-Roboter-Interaktion : Teilprojekt: Functional Safety : Schlussbericht : Laufzeit des Vorhabens: 01.08.2013-31.01.2016, Schlussbericht SafeAssistance, SafeAssista. doi:10.2314/GBV:870272594.

Hobbit (2020). HOBBIT – The mutual care robot. Available at: http://hobbit.acin.tuwien.ac.at/ [Accessed December 8, 2021].

Hochschule Bonn-Rhein-Sieg (2016). EmoRobot. Available at: https://www.h-brs.de/de/emorobot [Accessed December 7, 2021].

Hochschule Karlsruhe Technik und Wirtschaft (2020). BMBF-Projekt: PETRA – Personen-Transfer Roboter-Assistent. Available at: https://www.h-ka.de/iaf/petra [Accessed December 8, 2021].

Hochschule Ravensburg-weingarten (2020). RobotKoop. Available at: https://www.rwu.de/news-medien/aktuelles/pressemitteilungen/roboter-kurt-soll-im-alltag-helfen [Accessed December 7, 2021].

Hu, J., Edsinger, A., Lim, Y.-J., Donaldson, N., Solano, M., Solochek, A., et al. (2011). An advanced medical robotic system augmenting healthcare capabilities-robotic nursing assistant. in *2011 IEEE international conference on robotics and automation*, 6264–6269.

Huijnena, C., Lexisa, M., and de Wittea, L. (2015). Matching Robot KASPAR To ASD Therapy And Educational Goals. in *Conference Proceedings New Friends 2015*.

Hülsken-Giesler, M., and Remmers, H. (2020). *Robotische Systeme für die Pflege: Potenziale und Grenzen Autonomer Assistenzsysteme aus pflegewissenschaftlicher Sicht*. Vandenhoeck \& Ruprecht.

IEEE (2020). Robots. Your Guide to the World of Robotics: PR2. Available at: https://robots.ieee.org/robots/pr2/ [Accessed December 7, 2021].

IEEE SPECTRUM (2012). iRobot and InTouch Health Announce RP-VITA Telemedicine Robot. Available at: https://spectrum.ieee.org/automaton/robotics/medical-robots/irobot-and-intouch-health-announce-rpvita-telemedicine-robot [Accessed December 8, 2021].

Ihsen, S., Schneider, W., and Scheibl, K. (2012). Interdisciplinary research collaborations and learning processes of engineers and social scientists during the development of a robot for seniors in Europe. *World Trans. Eng. Technol. Educ.* 10.

Innophys (2020). Muscle Suit. Available at: https://innophys.jp/en/ [Accessed December 8, 2021].

Institute for Systems and Robotics | LISBOA (2020). AHA Project – Using games to improve health. Available at: https://welcome.isr.tecnico.ulisboa.pt/aha-project/ [Accessed December 8, 2021].

Irgenfried, Stephan and Schneider, J. (2016). MAID - Mobilitätsassistent zur Unterstützung bewegungseingeschränkter Personen : Teilvorhaben: Zustands- und intentionsabhängige Steuerung des Mobilitätsassistenten : Abschlussbericht zum Verbundvorhaben. doi:10.2314/GBV:871706237.

Ishiguro, H., Ono, T., Imai, M., Maeda, T., Kanda, T., and Nakatsu, R. (2001). Robovie: an interactive humanoid robot. *Ind. Robot An Int. J.*

Isken, M., Vester, B., Frenken, T., Steen, E.-E., Brell, M., and Hein, A. (2011). Enhancing Mobile Robots’ Navigation through Mobility Assessments in Domestic Environments. *Ambient Assist. Living*, 223–238. doi:10.1007/978-3-642-18167-2_16.

Ivlev, Oleg and Wilkening, André and Stöppler, Henning and Baiden, David and Wang, Chen and Xu, Chunya and Weishaupt, Mark and Sorgenicht, E. (2017). BMBF-Verbundprojekt SE_BURG - Intuitiv bedienbare bewegungsunterstützende Robotergeräte mit Selbstanpassung an Nutzer : Teilvorhaben: Soft-Antriebe und Regelung : Schlussbericht : Projektlaufzeit: 01.01.2014 bis 31.12.2016, Teilvorhaben: Soft-Antriebe und. doi:10.2314/GBV:89881149X.

Iwata, H., and Sugano, S. (2009). Design of human symbiotic robot TWENDY-ONE. in *2009 IEEE International Conference on Robotics and Automation*, 580–586.

Jacobs, T., and Graf, B. (2012). Practical evaluation of service robots for support and routine tasks in an elderly care facility. in *2012 IEEE Workshop on Advanced Robotics and its Social Impacts (ARSO)*, 46–49.

Jahn, E., Krause, J., and Müller, M. (2019). Intuitive Interaktion mit kooperativen Assistenzrobotern für das 3. und 4. Lebensalter (KoBo34): Evaluation von Bedürfnissen und Technikaffinität der Endnutzer/innen. *Mensch und Comput. 2019-Workshopband*.

Jardón, A., Gil, Á. M., de la Peña, A. I., Monje, C. A., and Balaguer, C. (2011). Usability assessment of ASIBOT: a portable robot to aid patients with spinal cord injury. *Disabil. Rehabil. Assist. Technol.* 6, 320–330.

Jason Maderer (2018). Robot Teaches Itself How to Dress People. Available at: https://news.gatech.edu/news/2018/05/14/robot-teaches-itself-how-dress-people [Accessed December 8, 2021].

Jeong, S., Breazeal, C., Logan, D., and Weinstock, P. (2018). Huggable: the impact of embodiment on promoting socio-emotional interactions for young pediatric inpatients. in *Proceedings of the 2018 CHI Conference on Human Factors in Computing Systems*, 1–13.

Jibo (2020). Meet Jibo. Available at: https://jibo.com/ [Accessed December 7, 2021].

Josué, F. P., Jorge, D. P., Beltran, M., and Jorge, M. C. (2015). NEW METHOD TO ATTACH WEARABLE ELECTRONICS TO CLOTHS. 39–42.

Keiser, Thomas and Hoppe, A. (2018). REHATHESE - Orthopädische Rehabilitation der unteren Extremitäten durch aktive Orthesen, “User Interface und Schnittstellen zur Rehabilitationsoptimierung”: Schlussbericht zum Förderprojekt : 01.04.2015 bis 31.12.2017. doi:10.2314/GBV:1663545588.

Kersten, A., and Brukamp, K. (2018). Robotik für die klinische Frühmobilisation.

KINOVA (2020a). Eating devices. Available at: https://www.kinovarobotics.com/en/assistive-technologies/column-a3/eating-devices [Accessed December 7, 2021].

KINOVA (2020b). KINOVA JACO Assistive robotic arm. Available at: https://www.kinovarobotics.com/en/products/assistive-technologies/kinova-jaco-assistive-robotic-arm [Accessed December 7, 2021].

KIT. Karlsruhe Institute of Technology (2020). RoSylerNT: Learning and robot-based systems for neuro-muscular training. Available at: https://www.ipr.kit.edu/english/315_2626.php [Accessed December 8, 2021].

Kittmann, R., Fröhlich, T., Schäfer, J., Reiser, U., Weißhardt, F., and Haug, A. (2015). Let me Introduce Myself: I am Care-O-bot 4, a Gentleman Robot. in *Mensch und Computer 2015 – Proceedings*, eds. S. Diefenbach, N. Henze, and M. Pielot (Berlin: De Gruyter Oldenbourg), 223–232.

Klinikum Stadt Soest (2016). Verbundprojekt: Oberkörperorthese zur Entlastung des Pflegepersonals bei körperlich belastenden Pflegeabläufen : CareJack : Teilvorhaben: Erarbeitung und Testung von Orthese-Demonstratoren unter pflegerische, praxisnahe Bedingungen : Projektlaufzeit: 01.1. doi:10.2314/GBV:876391498.

Koch, Christian and Haas, Hans-Dieter and Henni-Mansour, Karim and Fischer, M. (2017). Teilvorhaben Konstruktion und Sicherheit : Schlussbericht, Verbundvorhaben SE-BURG: Intuitiv bedienbare bewegungsunterstützende Robotergeräte mit Selbstanpassung an Nutzer, Teilvorhaben: Konstruktion und Sicherheit, SE_BURG Schlussbericht, Joint research. doi:10.2314/GBV:897259742.

Kolling, Thorsten and Klein, Barbara and Knopf, Monika and Oswald, Frank and Pantel, J. (2015). Verbundprojekt: “Chronische Krankheit, Funktionserhalt und Funktionsverluste im Alter - soziale und emotionale Ansprache durch Technik - \(ERimAlter\)”: Teilvorhaben: Methodenentwicklung und Evaluation zur Untersuchung emotionaler Ansprache durch Technik. doi:10.2314/GBV:851021107.

Kostelnik, J. (2016). Oberkörperorthese zur Entlastung des Pflegepersonals bei körperlich belastenden Pflegeabläufen - CareJACK : Schlussbericht : Laufzeit des Vorhabens: 01.10.2012 bis 31.12.2015: Titel des Teilvorhabens: WE-CIS Clip-In Starrflex, CareJack - WE RaS. doi:10.2314/GBV:870918176.

Kozima, H., Michalowski, M. P., and Nakagawa, C. (2009). Keepon. *Int. J. Soc. Robot.* 1, 3–18.

Kozima, H., Nakagawa, C., and Yasuda, Y. (2005). Interactive robots for communication-care: A case-study in autism therapy. in *ROMAN 2005. IEEE International Workshop on Robot and Human Interactive Communication, 2005.*, 341–346.

Kozima, H., Nakagawa, C., Yasuda, Y., and Kosugi, D. (2004). A toy-like robot in the playroom for children with developmental disorder. in *Proc. Int. Conf. Develop. Learning*, 188–189.

Krischak, Gert and Knapp, Sebastian and Knoll, A. (2017). Verbundprojekt: Intuitiv bedienbare bewegungsunterstützende Robotergeräte mit Selbstanpassung an Nutzer - SE_BURG : Teilvorhaben: Klinische Anwendung : Abschlussbericht : Laufzeit des Vorhabens: 01.01.2014-31.12.2016, Berichtszeitraum: 01.01.2014-31.12.20. doi:10.2314/GBV:896159647.

Krishnamurthy, B., and Evans, J. (1992). HelpMate: A robotic courier for hospital use. in *[Proceedings] 1992 IEEE International Conference on Systems, Man, and Cybernetics*, 1630–1634.

Kuhlmann, A., Reuter, V., Schramek, R., Dimitrov, T., Görnig, M., Matip, E.-M., et al. (2018). OurPuppet--Pflegeunterstützung mit einer interaktiven Puppe für pflegende Angehörige. *Z. Gerontol. Geriatr.* 51, 3–8.

Kuschan, J., Goppold, J.-P., Schmidt, H., and Krueger, J. (2018). PowerGrasp: Concept for a novel Soft-Robotic Arm Support System. in *ISR 2018; 50th International Symposium on Robotics*, 1–6.

Kuschan, J., Schmidt, H., and Krueger, J. (2016). Improved ergonomics via an intelligent movement and gesture detection jacket. in *Proceedings of ISR 2016: 47st International Symposium on Robotics*, 1–6.

Kuschan, J., Schmidt, H., and Krüger, J. (2017). Analysis of ergonomic and unergonomic human lifting behaviors by using Inertial Measurement Units. *Curr. Dir. Biomed. Eng.* 3, 7–10.

Kuzmicheva, O., Krebs, U., Martinez, S. F., Graeser, A., Blümke, C., and Steinhagen-Thiessen, E. (2015). MOPASS--Mobile Robotic System for Individualized Gait Rehabilitation.

Kuzmicheva, O., Martinez, S. F., Krebs, U., Spranger, M., Moosburner, S., Wagner, B., et al. (2016). Overground robot based gait rehabilitation system MOPASS-overview and first results from usability testing. in *2016 IEEE International Conference on Robotics and Automation (ICRA)*, 3756–3763.

Larsson, L. (2018). Verbundprojekt \"Orthopädische Rehabilitation der unteren Extremitäten durch aktive Orthesen - REHATHESE\"; Teilprojekt \"Energieeffizientes Antriebskonzept, Batteriemanagementsystem und Smart Home Bridge\" : Abschlussbericht : Laufzeit: 01.04.2015-31.05. doi:10.2314/KXP:1665889659.

Lee, J., Kim, J.-Y., Park, I.-W., Cho, B.-K., Kim, M., Kim, I., et al. (2006). Development of a humanoid robot platform HUBO FX-1. in *2006 SICE-ICASE International Joint Conference*, 1190–1194.

Lee, S., Noh, H., Lee, J., Lee, K., and Lee, G. G. (2010). Cognitive effects of robot-assisted language learning on oral skills. in *Second Language Studies: Acquisition, Learning, Education and Technology*.

Li, Z., Moran, P., Dong, Q., Shaw, R. J., and Hauser, K. (2017). Development of a tele-nursing mobile manipulator for remote care-giving in quarantine areas. *Proc. - IEEE Int. Conf. Robot. Autom.*, 3581–3586. doi:10.1109/ICRA.2017.7989411.

Life Science robotics (2020). Meet ROBERT. Available at: https://www.lifescience-robotics.com/meet-robert/ [Accessed December 8, 2021].

Löffler, D., Dörrenbächer, J., Welge, J., and Hassenzahl, M. (2020). Hybridity as design strategy for service robots to become domestic products. in *Extended Abstracts of the 2020 CHI Conference on Human Factors in Computing Systems*, 1–8.

Lowet, D., Isken, M., Lee, W., Van Heesch, F., and Eertink, E. (2012). Robotic telepresence for 24/07 remote assistance to elderly at home. *Soc. Robot. Telepresence*, 17.

Luperto, M., Monroy, J., Ruiz-sarmiento, J. R., Moreno, F., Basilico, N., Gonzalez-jimenez, J., et al. (2019a). Towards Long-Term Deployment of a Mobile Robot for at-Home Ambient Assisted Living of the Elderly. in *2019 European Conference on Mobile Robots (ECMR)* (IEEE), 1--6.

Luperto, M., Romeo, M., Lunardini, F., Basilico, N., Abbate, C., Jones, R., et al. (2019b). Evaluating the Acceptability of Assistive Robots for Early Detection of Mild Cognitive Impairment. in *2019 IEEE/RSJ International Conference on Intelligent Robots and Systems (IROS)* (IEEE), 1257–1264.

Mahrouche, I. (2015). Robotic Nursing & Caring-Study Example: Robotic Nursing Assistant (RoNA) System. in *International Conference on Industrial Engineering and Operations Management (IEOM), Dubai, United Arab Emirates, Mar*, 3–5.

Maple, C., Yue, Y., Li, D., and Bochenkov, A. (2012). Case study: Multi-role shadow robotic system for independent living. *Proc. 2012 Int. Conf. High Perform. Comput. Simulation, HPCS 2012*, 415. doi:10.1109/HPCSim.2012.6266950.

Marsi-Bionics (2022). Marsi Bionics develops the most innovative technology for gait trainning. Available at: https://www.marsibionics.com/en/ [Accessed January 19, 2022].

Martinez, S. F., Kuzmicheva, O., and Graeser, A. (2016). Joint trajectory generation and control for overground robot-based gait rehabilitation system MOPASS. in *Proceedings of the 7th Augmented Human International Conference 2016*, 1–4.

Martini, E., Livolsi, C., Pergolini, A., Arnetoli, G., Doronzio, S., Giffone, A., et al. (2020). Lower-limb amputees can reduce the energy cost of walking when assisted by an Active Pelvis Orthosis. in *2020 8th IEEE RAS/EMBS International Conference for Biomedical Robotics and Biomechatronics (BioRob)*, 809–815. doi:10.1109/biorob49111.2020.9224417.

Martins, S., and Dias, J. (2020). “The GrowMeUp Project and the Applicability of Action Recognition Techniques,” in *Third workshop on recognition and action for scene understanding (REACTS). Ruiz de Aloza*, 1–14.

Matsusaka, Y., Fujii, H., Okano, T., and Hara, I. (2009). Health exercise demonstration robot TAIZO and effects of using voice command in robot-human collaborative demonstration. in *RO-MAN 2009-The 18th IEEE International Symposium on Robot and Human Interactive Communication*, 472–477.

Mayer, P., Beck, C., and Panek, P. (2012). Examples of multimodal user interfaces for socially assistive robots in ambient assisted living environments. in *2012 IEEE 3rd International Conference on Cognitive Infocommunications (CogInfoCom)*, 401–406.

Mayer, P., and Panek, P. (2013). A social assistive robot in an intelligent environment. *Biomed. Eng. Tech.* 58, 000010151520134240.

MEDICA Magazine (2019). Training partner robot – This is the rehabilitation oft he future. Available at: https://www.medica-tradefair.com/en/MediaCenter/Older_Background_Reports/Background_Reports_2019/Training_partner_robot_%5C%E2%5C%80%5C%93_This_is_the_rehabilitation_of_the_future [Accessed June 12, 2020].

Meschtscherjakov, A., De Ruyter, B., Fuchsberger, V., Murer, M., and Tscheligi, M. (2016). “Persuasive Technology: 11th International Conference, PERSUASIVE 2016, Salzburg, Austria, April 5-7, 2016, Proceedings,” in (Springer), 317–318.

MetraLabs mobile robots (2020). SCITOS G5 The All-rounder. Available at: https://www.metralabs.com/mobiler-roboter-scitos-g5/ [Accessed December 7, 2021].

Mike Topping (1998). An overview of Handy 1, a rehabilitation robot for the severely disable. Available at: https://www.dinf.ne.jp/doc/english/Us_Eu/conf/csun_99/session0059.html [Accessed December 8, 2021].

MobIPaR-Projekt (2020). Das Projekt: MobIPaR. Available at: https://www.mobipar-projekt.de/index.html [Accessed December 7, 2021].

MOBOT (2013). MOBOT_Flyer. Available at: http://www.mobot-project.eu/userfiles/downloads/DisseminationMaterial/MOBOT_Flyer_v1-7.pdf [Accessed June 1, 2020].

Moreno, P., Nunes, R., Figueiredo, R., Ferreira, R., Bernardino, A., Santos-Victor, J., et al. (2016). Vizzy: A humanoid on wheels for assistive robotics. in *Robot 2015: Second Iberian Robotics Conference*, 17–28.

Moritz, Christoph and Hahn, M. (2016). Oberkörperorthese zur Entlastung des Pflegepersonals bei körperlich belastenden Pflegeabläufen : Teilvorhaben: Mechatronische Systementwicklung : fachlicher Abschlussbericht zum Verbundprojekt: CareJack : Berichtszeitraum: 01.10.2012-31.12.2015. doi:10.2314/GBV:870664751.

MORPHIA (2020). Zielstellung des Projekts MORPHIA. Available at: http://www.morphia-projekt.de/ [Accessed December 8, 2021].

MoveCare (2020). MULTIPLE-ACTORS VIRTUAL EMPATHIC CAREGIVER FOR THE ELDER. Available at: http://www.movecare-project.eu/ [Accessed January 14, 2022].

MTI-engage (2020). Willkommen auf der Homepage von MTI-engAge. Available at: https://www.mti-engage.tu-berlin.de/ [Accessed December 7, 2021].

Mukai, T., Hirano, S., Nakashima, H., Kato, Y., Sakaida, Y., Guo, S., et al. (2010). Development of a nursing-care assistant robot RIBA that can lift a human in its arms. in *2010 IEEE/RSJ International Conference on Intelligent Robots and Systems*, 5996–6001.

Mularczyk, P. (2016). Verbundprojekt: Oberkörperorthese zur Entlastung des Pflegepersonals bei körperlich belastenden Pflegeabläufen - CareJack : Schlussbericht : Projektlaufzeit: 01.10.2012-31.12.2015. doi:10.2314/GBV:883577720.

Müller, F. (2018). Verbundprojekt “Orthopädische Rehabilitation der unteren Extremitäten durch aktive Orthesen - REHATHESE”; Teilprojekt “Untersuchung, Bewertung und Optimierung der technologischen und therapeutischen Lösungsansätze” : Abschlussbericht : Laufzeit: 01.04.201. doi:10.2314/KXP:1665860553.

Müller, S., Schaffernicht, E., Scheidig, A., Böhme, H.-J., and Gross, H.-M. (2007). Are you still following me? in *EMCR*.

Muscle (2020). ROBOHelper SASUKE. Available at: https://www.musclerobo.com/ [Accessed December 7, 2021].

Mutlu, B., and Forlizzi, J. (2008). Robots in organizations: the role of workflow, social, and environmental factors in human-robot interaction. in *2008 3rd ACM/IEEE International Conference on Human-Robot Interaction (HRI)*, 287–294.

Naotunna, I., Perera, C. J., Sandaruwan, C., Gopura, R., and Lalitharatne, T. D. (2015). Meal assistance robots: A review on current status, challenges and future directions. in *2015 IEEE/SICE International Symposium on System Integration (SII)*, 211–216.

Naroska, E., Schmitz, S., Bolten, T., Lee, C.-W., Tsai, P.-C., and Ruan, S.-J. (2018). Project OurPuppet: A system to support people with dementia and their caregiving relatives at home. in *2018 IEEE International Conference on Consumer Electronics (ICCE)*, 1–4.

NASA (2003). A Robot to Help Make the Rounds. Available at: https://spinoff.nasa.gov/spinoff2003/hm_4.html [Accessed December 7, 2021].

Niechwiadowicz, K., and Khan, Z. (2008). Robot based logistics system for hospitals-survey. in *IDT Workshop on interesting results in computer science and engineering*.

of Robotics, I. F. of R. (2016). Introduction into Service Robots.

OFFIS (2013). Florence Multi Purpose Robot for Ambient Assisted Living. Available at: https://www.offis.de/en/offis/project/florence.html [Accessed January 17, 2022].

Onishi, M., Luo, Z., Odashima, T., Hirano, S., Tahara, K., and Mukai, T. (2007). Generation of human care behaviors by human-interactive robot RI-MAN. in *Proceedings 2007 IEEE International Conference on Robotics and Automation*, 3128–3129.

OTW Orthopädietechnik Winkler (2016). Öffentlicher Schlussbericht CareJack : 01.10.2012-31.12.2015. doi:10.2314/GBV:867053380.

OurPuppet (2019). About OurPuppet. Available at: https://www.ourpuppet.de/en/about-ourpuppet/25 [Accessed December 8, 2021].

Pal Robotics (2022a). EnrichMe. Available at: https://pal-robotics.com/es/proyectos-colaborativos/enrichme/ [Accessed January 14, 2022].

Pal Robotics (2022b). GrowMeUp. Available at: https://pal-robotics.com/es/proyectos-colaborativos/growmeup/ [Accessed January 14, 2022].

Panasonic (2015). Panasonic Autonomous Delivery Robots – HOSPI- Aid Hospital Operations at Changi General Hospital. Available at: https://news.panasonic.com/global/topics/2015/44009.html [Accessed December 7, 2021].

Panasonic (2019). Autonomous Mobility Robot for More Secure and Comfortable Life. Available at: https://news.panasonic.com/global/stories/2019/69861.html [Accessed December 7, 2021].

Panasonic (2020). Resyone Plus. Robotic Care Bed/Wheelchair. Available at: https://www.panasonic.oa.hk/english/products/age-free-product/resyone-plus/xpn-s10601hk.aspx [Accessed December 8, 2021].

PARO Robots U.S., I. (2014). PARO, Therapeutic Robot. Available at: http://www.parorobots.com/ [Accessed May 27, 2020].

Peleka, G., Kargakos, A., Skartados, E., Kostavelis, I., Giakoumis, D., Sarantopoulos, I., et al. (2018). RAMCIP-a service robot for MCI patients at home. in *2018 IEEE/RSJ International Conference on Intelligent Robots and Systems (IROS)*, ed. IEEE, 1--9.

Personal Robotics Lab. Imperial College London (2020). Personalized Robot-Assisted Dressing. Available at: http://www.imperial.ac.uk/personal-robotics/research/personalized-_robot_assisted_dressing/ [Accessed December 7, 2021].

Personal Robots Group (2015). Huggable. Available at: http://robotic.media.mit.edu/portfolio/huggable/ [Accessed December 8, 2021].

Pilla, A., Trigili, E., McKinney, Z., Fanciullacci, C., Malasoma, C., Posteraro, F., et al. (2020). Robotic Rehabilitation and Multimodal Instrumented Assessment of Post-stroke Elbow Motor Functions—A Randomized Controlled Trial Protocol. *Front. Neurol.* 11, 1–11. doi:10.3389/fneur.2020.587293.

Pillohealth (2020). Meet Pillo, The Home Health Companion. Available at: https://pillohealth.com/devices [Accessed December 8, 2021].

Pitsch, K., and Koch, B. (2010). How infants perceive the toy robot pleo. an exploratory case study on infant-robot-interaction. in *Second International Symposium on New Frontiers in Human-Robot-Interaction (AISB)*.

PLEOrb (2012). What is PLEO rb. Available at: https://www.pleoworld.com/pleo_rb/eng/lifeform.php [Accessed December 7, 2021].

Pollack, M. E., Brown, L., Colbry, D., Orosz, C., Peintner, B., Ramakrishnan, S., et al. (2002). Pearl: A mobile robotic assistant for the elderly. in *AAAI workshop on automation as eldercare*, 85–91.

Portugal, D., Alvito, P., Christodoulou, E., Samaras, G., and Dias, J. (2019). A Study on the Deployment of a Service Robot in an Elderly Care Center. *Int. J. Soc. Robot.* 11, 317–341. doi:10.1007/s12369-018-0492-5.

Prassler, Erwin and Ziegler, Sven and Bleses, Helma M. and Füller, M. (2017). Emotionen stimulierende Assistenzroboter in der Pflege und Betreuung dementiell erkrankter Menschen in der stationären Langzeitpflege \(EmoRobot\) : gemeinsamer Abschlussbericht des Verbundvorhabens der Hochschulen Fulda und Bonn-Rhein-Sieg : Berichtszeit. doi:10.2314/GBV:893736007.

Prof. Dympna Casey (2019). MARIO Project. Available at: http://www.mario-project.eu/PORTAL/ [Accessed December 7, 2021].

Pulido, J. C., González, J. C., Suárez-Mej\’\ias, C., Bandera, A., Bustos, P., and Fernández, F. (2017). Evaluating the child--robot interaction of the NAOTherapist platform in pediatric rehabilitation. *Int. J. Soc. Robot.* 9, 343–358.

Puyuelo-Quintana, G., Cano-de-la-Cuerda, R., Plaza-Flores, A., Garces-Castellote, E., Sanz-Merodio, D., Goñi-Arana, A., et al. (2020). A new lower limb portable exoskeleton for gait assistance in neurological patients : a proof of concept study. *J. Neuroeng. Rehabil.* 17, 1–16.

R.-T. C. C. for Human-Interactive Robot Research(RTC) (2020). RIBA: World’s first robot that can lift up a human in its arms. Available at: http://rtc.nagoya.riken.jp/RIBA/index-e.html [Accessed December 8, 2021].

R. Robotics (2020a). ReWalkTM Personal 6.0. Available at: https://rewalk.com/rewalk-personal-3/ [Accessed December 7, 2021].

R. Robotics (2020b). The ReStoreTM Soft Exo-Suit. Available at: https://rewalk.com/restore-exo-suit/ [Accessed December 8, 2021].

Ramdani, N., Panayides, A., Karamousadakis, M., Mellado, M., Lopez, R., Christophorou, C., et al. (2019). A safe, efficient and integrated indoor robotic fleet for logistic applications in healthcare and commercial spaces: The endorse concept. *Proc. - IEEE Int. Conf. Mob. Data Manag.* 2019-June, 425–430. doi:10.1109/MDM.2019.000-8.

Rane, P., Mhatre, V., and Kurup, L. (2014). Study of a home robot: Jibo. *Int. J. Eng. Res. Technol.* 3, 490–493.

Reeti (2020). "Reeti: an expressive and communicating robot. Available at: http://reeti.fr/index.php/en/ [Accessed December 8, 2021].

Rehrl, T., Geiger, J., Golcar, M., Gentsch, S., Knobloch, J., Rigoll, G., et al. (2014). “The robot ALIAS as a database for health monitoring for elderly people,” in *Ambient Assisted Living* (Springer), 225–245.

ReHyb (2022). ABOUT THE REHYB PROJECT. Available at: https://rehyb.eu/ [Accessed January 14, 2022].

Reichel, S., Weidemann, A., Compagna, D., and Deimel, R. (2017). Partizipatives Entwerfen zukünftiger Roboter. *Inform. 2017*.

Reif, Wolfgang and Hoffmann, A. (2016). SafeAssistance : Schlussbericht zum Teilvorhaben Umgebungsmodellierung \& Reaktionsstrategien im Verbundprojekt SafeAssistance - Intelligente Hinderniserkennung mit kapazitiven Sensoren zur sicheren Mensch-Roboter-Interaktion. doi:10.2314/GBV:877008264.

Resinger, R. (2017). SE_BURG: BMBF-Verbundprojekt Intuitiv bedienbare bewegungsunterstützende Robotergeräte mit Selbstanpassung an Nutzer : Teilvorhaben: Materialforschung, Herstellungstechnologien und Untersuchungen für dynamische Polsterungen, Schlussbericht SE_BURG, Teilvo. doi:10.2314/GBV:885350340.

Ritschel, H., and André, E. (2017). Real-time robot personality adaptation based on reinforcement learning and social signals. in *Proceedings of the companion of the 2017 acm/ieee international conference on human-robot interaction*, 265–266.

Ritschel, H., Seiderer, A., Janowski, K., Aslan, I., and André, E. (2018). Drink-o-mender: An adaptive robotic drink adviser. in *Proceedings of the 3rd International Workshop on Multisensory Approaches to Human-Food Interaction*, 1–8.

ROBINA (2020). ROBINA | Home. Available at: https://projekt-robina.de/ [Accessed December 8, 2021].

Robot Center (2020). Texai. Available at: https://www.robotcenter.co.uk/products/texai [Accessed December 8, 2021].

Robotic und produktion (2019). Roboter in der Pflege. Available at: https://www.robotik-produktion.de/allgemein/roboter-in-der-pflege/ [Accessed December 7, 2021].

Robotics today (2003). Dr.Robot. Available at: https://www.roboticstoday.com/robots/dr-robot-description [Accessed December 7, 2021].

Robotics today (2006a). Huggable. Available at: https://www.roboticstoday.com/robots/huggable-description [Accessed December 7, 2021].

Robotics today (2006b). RI-MAN. Available at: https://www.roboticstoday.com/robots/ri-man-description [Accessed December 7, 2021].

Robotics today (2007). HUBO FX-1. Available at: https://www.roboticstoday.com/robots/hubo-fx-1-description [Accessed December 7, 2021].

Robotics today (2008). Keio Robot. Available at: https://www.roboticstoday.com/robots/keio-robot [Accessed December 8, 2021].

Robotics today (2009a). Care Robot Yurina. Available at: https://www.roboticstoday.com/robots/care-robot-yurina-description [Accessed December 7, 2021].

Robotics today (2009b). ROLA. Available at: https://www.roboticstoday.com/robots/rola-description [Accessed December 8, 2021].

Robotics today (2009c). Taizo. Available at: https://www.roboticstoday.com/robots/taizo [Accessed December 7, 2021].

Robotics today (2010a). ATOM 7xp. Available at: https://www.roboticstoday.com/robots/atom-7xp-description [Accessed December 8, 2021].

Robotics today (2010b). Care-O-bot 3. Available at: https://www.roboticstoday.com/robots/care-o-bot-3-description [Accessed December 8, 2021].

Robotics today (2010c). Core. Available at: https://www.roboticstoday.com/robots/core-description [Accessed December 7, 2021].

Robotics today (2010d). Family Nanny Robot. Available at: https://www.roboticstoday.com/robots/family-nanny-robot-description [Accessed December 8, 2021].

Robotics today (2010e). Fujitsu Teddy Bear. Available at: https://www.roboticstoday.com/robots/fujitsu-teddy-bear-description [Accessed December 7, 2021].

Robotics today (2010f). HuiHui. Available at: https://www.roboticstoday.com/robots/huihui-description [Accessed December 7, 2021].

Robotics today (2010g). Robotic Bed. Available at: https://www.roboticstoday.com/robots/robotic-bed-description [Accessed December 7, 2021].

Robotics today (2010h). Robovie. Available at: https://www.roboticstoday.com/robots/robovie-r3unr-description [Accessed December 8, 2021].

Robotics today (2013). Eva. Available at: https://www.roboticstoday.com/robots/eva-description [Accessed December 8, 2021].

Robotics today (2014). HelloSpoon. Available at: https://www.roboticstoday.com/robots/hellospoon-description [Accessed December 8, 2021].

Robotics today (2015a). HOSPI. Available at: https://www.roboticstoday.com/robots/hospi-description [Accessed December 8, 2021].

Robotics today (2015b). Jibo. Available at: https://www.roboticstoday.com/robots/jibo-description [Accessed December 8, 2021].

Robotics Today (1997). Robota Dolls. Available at: https://www.roboticstoday.com/robots/robota-dolls-description [Accessed December 7, 2021].

Robotics Today (2008). Rhoni. Available at: https://www.roboticstoday.com/robots/rhoni [Accessed December 8, 2021].

Robotics Today (2010a). EngKey. Available at: https://www.roboticstoday.com/robots/engkey [Accessed December 7, 2021].

Robotics Today (2010b). MERO. Available at: https://www.roboticstoday.com/robots/mero [Accessed December 8, 2021].

RobotKoop (2020). Kooperative Interaktion und Zielverhandlung mit lernenden autonomen Robotern. Available at: https://www.robotkoop.de/ [Accessed December 8, 2021].

RobotShop (2020). Reeti Expressive Humanoir Development Platform. Available at: https://www.robotshop.com/en/reeti-humanoid-development-platform.html [Accessed June 5, 2020].

ROBOTSVOICE (2013). Reeti. Available at: http://www.robotsvoice.com/reeti/ [Accessed December 7, 2021].

Robtos.nu (2020). Zeno robot. Available at: https://robots.nu/en/robot/zeno-robot [Accessed December 7, 2021].

Robtos4autism (2020). Meet Milo. Available at: https://www.robokind.com/robots4autism/meet-milo [Accessed December 8, 2021].

RoPHa (2020). Robuste Perzeption für die interaktive Unterstützung älterer Nutzer bei Handhabungsaufgaben im häuslichen Umfeld. Available at: https://www.ropha-projekt.de/ [Accessed December 8, 2021].

Roreas (2017). roreas. Robotischer Reha Assistant. Available at: http://www.roreas.org/ [Accessed December 7, 2021].

Rosenberg, A. (2016). SafeAssistance - intelligente Hinderniserkennung mit kapazitiven Sensoren zur sicheren Mensch-Roboter-Interaktion : Abschlussbericht, SafeAssistance - intelligent obstacle detection by capacitive sensors for a safe human robot interaction. doi:10.2314/GBV:876458258.

Salatino, C., Gower, V., Ghrissi, M., Tapus, A., Wieczorowska-tobis, K., Suwalska, A., et al. (2016). “The EnrichMe Project A Robotic Solution for Independence and Active Aging of Elderly,” in *Computers Helping People with Special Needs* (Springer International Publishing), 326–334. doi:10.1007/978-3-319-41264-1.

Salichs, E., Castro-González, Á., Malfaz, M., and Salichs, M. A. (2016). Mini: a social assistive robot for people with mild cognitive impairment. *New Friends*, 31–32.

Salvador, M. J., Silver, S., and Mahoor, M. H. (2015). An emotion recognition comparative study of autistic and typically-developing children using the zeno robot. in *2015 IEEE International Conference on Robotics and Automation (ICRA)*, 6128–6133.

Schaeffer, C., and May, T. (1999). Care-o-bot-a system for assisting elderly or disabled persons in home environments. *Assist. Technol. Threshold new millenium*, 3.

Schroeter, C., Mueller, S., Volkhardt, M., Einhorn, E., Huijnen, C., van den Heuvel, H., et al. (2013). Realization and user evaluation of a companion robot for people with mild cognitive impairments. in *2013 IEEE International Conference on robotics and automation*, 1153–1159.

SECOM (2020). My Spoon. Available at: https://www.secom.co.jp/english/myspoon/ [Accessed May 19, 2020].

SeRoDi. Servicerobotik in der Pflege (2020). Über SeRoDi. Available at: http://www.serodi.de/?page_id=5 [Accessed December 8, 2021].

Servaty, R., Möhler, R., Kersten, A., Brukamp, K., and Müller, M. (2018). Barriers and facilitators of implementing robotic systems in nursing care. *training* 13, 14.

Sgorbissa, A., Saffiotti, A., Battistuzzi, L., Menicatti, R., Pecora, F., and Papadopoulos, I. (2019). CARESSES : The Flower that Taught Robots about Culture. 8555.

Siciliano, B., and Khatib, O. (2016). *Springer handbook of robotics*. Springer.

Softbank Robotics (2020a). Healthcare. Available at: https://www.softbankrobotics.com/emea/en/industries/healthcare [Accessed December 7, 2021].

Softbank Robotics (2020b). Pepper. Available at: https://us.softbankrobotics.com/pepper [Accessed December 7, 2021].

Softbank Robotics (2020c). Project ROMEO. Available at: https://projetromeo.com/ [Accessed May 22, 2020].

Song, K.-T., Tsai, C.-Y., Huang, F.-S., Hong, J.-W., Lin, C.-Y., Chen, C.-W., et al. (2008). Development of the robot of living aid: RoLA. in *2008 IEEE International Conference on Automation and Logistics*, 443–448.

Spexor (2016). Spinal exoskeletal robot for low back pain prevention and vocational reintegration. Available at: spexor.eu [Accessed January 17, 2022].

Stiehl, W. D., Breazeal, C., Han, K.-H., Lieberman, J., Lalla, L., Maymin, A., et al. (2006). “The huggable: a therapeutic robotic companion for relational, affective touch,” in *ACM SIGGRAPH 2006 emerging technologies*, 15--es.

Stoelen, M. F., Tejada, V. F., Jardón, A., Bonsignorio, F., and Balaguer, C. (2016). Adaptive aid on targeted robot manipulator movements in tele-assistance. *Paladyn, J. Behav. Robot.* 7.

Stollnberger, G., Moser, C., Beck, E., Zenz, C., Tscheligi, M., Szczesniak-Stanczyk, D., et al. (2014). Robotic systems in health care. in *2014 7th International Conference on Human System Interactions (HSI)*, 276–281.

SYMPARTNER (2018). Zielstellungen von SYMPARTNER. Available at: http://www.sympartner.de/ [Accessed December 7, 2021].

Takahashi, M., Suzuki, T., Cinquegrani, F., Sorbello, R., and Pagello, E. (2009). A mobile robot for transport applications in hospital domain with safe human detection algorithm. in *2009 IEEE International Conference on Robotics and Biomimetics (ROBIO)*, 1543–1548.

Tanioka, R., Sugimoto, H., Yasuhara, Y., Ito, H., Osaka, K., Zhao, Y., et al. (2019). Characteristics of transactive relationship phenomena among older adults, care workers as intermediaries, and the Pepper robot with care prevention gymnastics exercises. *J. Med. Investig.* 66, 46–49.

Tasaki, R., Kitazaki, M., Miura, J., and Terashima, K. (2015). Prototype design of medical round supporting robot “Terapio.” in *2015 IEEE International Conference on Robotics and Automation (ICRA)*, 829–834.

TechCrunch (2010). Videos:Health Care Robot Yurina. Available at: https://techcrunch.com/2010/08/13/videos-health-care-robot-yurina/ [Accessed December 8, 2021].

Technische Hochschule Rosenheim (2020). Projekt KoBo34. Available at: https://www.th-rosenheim.de/forschung-entwicklung/kompetenzfelder-und-projekte/gesundheit-soziales-u-paedagogik/kobo34/ [Accessed December 7, 2021].

The Future of Things (2020). Toyota i-foot Robot. Available at: https://thefutureofthings.com/5258-toyota-i-foot-robot/ [Accessed December 7, 2021].

Tokyo Skytree Town Campus (2020). Core. Available at: https://www.it-chiba.ac.jp/skytree/en/exhibitions/core/ [Accessed December 7, 2021].

Topping, M. (1995). The development of Handy 1, a robotic aid to independence for the severely disabled.

TUDelft (2020). Robot EVA. Available at: https://www.tudelft.nl/en/kennisvalorisatie/ontwikkeling-innovatie/innovation-projects/robot-eva/ [Accessed December 7, 2021].

Tufts University (2018). KIBO Robotics. A developmentally appropriate robotics kit for children aged 4-7. Available at: https://ase.tufts.edu/devtech/readyforrobotics/research.asp [Accessed December 8, 2021].

TWENDY-ONE (2007). Concept. Available at: http://www.twendyone.com/concept_e.html [Accessed December 7, 2021].

Universität Augsburg (2020). ForGenderCare. Available at: https://www.informatik.uni-augsburg.de/lehrstuehle/hcm/projects/external/ForGenderCare/ [Accessed June 5, 2020].

Universität Bremen (2015). MOPASS: Mobiles, dem Patienten angepasstes, robotergestütztes Gangrehabilitationssystem. Available at: http://www.iat.uni-bremen.de/sixcms/detail.php?id=1483 [Accessed December 7, 2021].

Universität Duisburg-Essen (2014). WiMi-Care. Available at: https://www.uni-due.de/wimi-care/ [Accessed December 7, 2021].

University of Hertfordshire (2020). Kaspar the social robot. Available at: https://www.herts.ac.uk/kaspar [Accessed December 8, 2021].

University of Pittsburgh (2020). PerMMA. Available at: https://www.herl.pitt.edu/research/permma [Accessed December 8, 2021].

Vincze, M., Zagler, W., Lammer, L., Weiss, A., Huber, A., Fischinger, D., et al. (2014). Towards a robot for supporting older people to stay longer independent at home. in *ISR/Robotik 2014; 41st International Symposium on Robotics*, 1–7.

Vital, J. P. M., Couceiro, M. S., Rodrigues, N. M. M., Figueiredo, C. M., and Ferreira, N. M. F. (2013). Fostering the NAO platform as an elderly care robot. in *2013 IEEE 2nd international conference on serious games and applications for health (SeGAH)*, 1–5.

Vital, J. P. M., Ferreira, N. M. F., and Valente, A. (2018). Nao robot as a domestic robot. in *Memorias de Congresos UTP*, 243–250.

Wagner, G. (2016). Abschlussbericht zum Verbundprojekt MAID - Mobilitätsassistent zur Unterstützung bewegungseingeschränkter Personen : Teilvorhaben: Kreislaufüberwachung, Final report on subproject \"Cardiovascular monitoring\" in the joint project \"MAID-Mobility AID for. doi:10.2314/GBV:872850552.

Wainer, J., Dautenhahn, K., Robins, B., and Amirabdollahian, F. (2014a). A pilot study with a novel setup for collaborative play of the humanoid robot KASPAR with children with autism. *Int. J. Soc. Robot.* 6, 45–65.

Wainer, J., Robins, B., Amirabdollahian, F., and Dautenhahn, K. (2014b). Using the humanoid robot KASPAR to autonomously play triadic games and facilitate collaborative play among children with autism. *IEEE Trans. Auton. Ment. Dev.* 6, 183–199.

Wang, H., Grindle, G. G., Candiotti, J., Chung, C., Shino, M., Houston, E., et al. (2012). The Personal Mobility and Manipulation Appliance (PerMMA): A robotic wheelchair with advanced mobility and manipulation. in *2012 Annual International Conference of the IEEE Engineering in Medicine and Biology Society*, 3324–3327.

Wilkinson, J. (PRIKEN) (2020). The strong robot with the gentle touch. Available at: https://www.riken.jp/en/news_pubs/research_news/pr/2015/20150223_2/ [Accessed December 8, 2021].

Willemse, C. J. A. M., and van Erp, J. B. F. (2019). Social touch in Human--robot interaction: Robot-initiated touches can induce positive responses without extensive prior bonding. *Int. J. Soc. Robot.* 11, 285–304.

Willow garage (2015). Texai Remote Presence System. Available at: http://www.willowgarage.com/pages/texai/overview [Accessed June 9, 2020].

Wimmer, Christian and Urquizar, Carlos and Hammer, Mikael and Saez, M. (2017). Autonomer Robotik Rollstuhl : Kurztitel: AuRoRoll : eingereicht im Anwendungsfeld: Informations- und Kommunikationstechnologie : Schlussbericht zum Themenfeld: Mensch-Technik-Interaktion für den demografischen Wandel : Laufzeit: 36 Monate, Abschlussberich. doi:10.2314/GBV:891757791.

Wolschke, Mirko and Liebach, Jana and Sommerfeld, Kamila and Smurawski, A. (2016). Verbundprojekt: Oberkörperorthese zur Entlastung des Pflegepersonals bei körperlich belastenden Pflegeabläufen - CareJack : Teilvorhaben: Erarbeitung und Testung von Bewegungsmodulen unter medizinischen Aspekten : Projektlaufzeit: 01.10.2012-31.12.2015. doi:10.2314/GBV:875709257.

Wood, L. J., Zaraki, A., Walters, M. L., Novanda, O., Robins, B., and Dautenhahn, K. (2017). The iterative development of the humanoid robot kaspar: An assistive robot for children with autism. in *International Conference on Social Robotics*, 53–63.

Worcester Polytechnic Institute (2022). Tele-Robotic Intelligent Nursing Assistant (TRINA). Available at: http://labs.wpi.edu/hiro/research/share-autonomous-nursing-robot/trina/.

Zeilfelder, J., Busch, T., Zimmermann, C., and Stork, W. (2018). A human-machine interface based on tongue and jaw movements. in *2018 IEEE Sensors Applications Symposium (SAS)*, 1–6.

Zhang, F., Cully, A., and Demiris, Y. (2017). Personalized robot-assisted dressing using user modeling in latent spaces. in *2017 IEEE/RSJ International Conference on Intelligent Robots and Systems (IROS)*, 3603–3610.

Zhang, F., Cully, A., and Demiris, Y. (2019). Probabilistic real-time user posture tracking for personalized robot-assisted dressing. *IEEE Trans. Robot.* 35, 873–888.

Zhang, T., Zhu, B., Lee, L., and Kaber, D. (2008). Service robot anthropomorphism and interface design for emotion in human-robot interaction. in *2008 IEEE International Conference on Automation Science and Engineering*, 674–679.

Zorabots (2020). NAO for Zora. Available at: https://www.zorabots.be/site/index.php/en/who-am-i [Accessed December 7, 2021].
